# A systematic safety pipeline for selection of T-cell receptors to enter clinical use

**DOI:** 10.1038/s41541-023-00713-y

**Published:** 2023-08-22

**Authors:** Zsofia Foldvari, Cathrine Knetter, Weiwen Yang, Thea Johanne Gjerdingen, Ravi Chand Bollineni, Trung The Tran, Fridtjof Lund-Johansen, Arne Kolstad, Kimberley Drousch, Robert Klopfleisch, Matthias Leisegang, Johanna Olweus

**Affiliations:** 1 Department of Cancer Immunology, Institute for Cancer Research, Oslo University Hospital Radiumhospitalet, Oslo, Norway; 2 Precision Immunotherapy Alliance, University of Oslo, Oslo, Norway; 3 Department of Immunology, University of Oslo and Oslo University Hospital, Oslo, Norway; 4 Department of Oncology, Oslo University Hospital Radiumhospitalet, Oslo, Norway; 5 Institute of Immunology, Charité - Universitätsmedizin Berlin, Berlin, Germany; 6 Institute of Veterinary Pathology, Freie Universität Berlin, Berlin, Germany; 7 David and Etta Jonas Center for Cellular Therapy, The University of Chicago, Chicago, IL USA; 8 German Cancer Consortium (DKTK), partner site Berlin, and German Cancer Research Center (DKFZ), Heidelberg, Germany

**Keywords:** Translational research, Cancer immunotherapy

## Abstract

Cancer immunotherapy using T cell receptor-engineered T cells (TCR-Ts) represents a promising treatment option. However, technologies for pre-clinical safety assessment are incomplete or inaccessible to most laboratories. Here, TCR-T off-target reactivity was assessed in five steps: (1) Mapping target amino acids necessary for TCR-T recognition, followed by (2) a computational search for, and (3) reactivity screening against, candidate cross-reactive peptides in the human proteome. Natural processing and presentation of recognized peptides was evaluated using (4) short mRNAs, and (5) full-length proteins. TCR-Ts were screened for recognition of unintended HLA alleles, and as proxy for off-target reactivity in vivo, a syngeneic, HLA-A*02:01-transgenic mouse model was used. Validation demonstrated importance of studying recognition of full-length candidate off-targets, and that the clinically applied 1G4 TCR has a hitherto unknown reactivity to unintended HLA alleles, relevant for patient selection. This widely applicable strategy should facilitate evaluation of candidate therapeutic TCRs and inform clinical decision-making.

## Introduction

T cells engineered to express tumor-specific T cell receptors (TCRs) can effectively redirect a patient’s immune response against tumor antigens. Adoptive transfer of TCR-engineered T cells (TCR-Ts) has already led to encouraging results, suggesting use of TCR-Ts in cancer treatment on a broad basis^1–3^. In addition to selecting appropriate target antigens and generating therapeutic TCRs, the preclinical analysis of TCRs is a critical bottleneck because it is essential to ensure safe and efficient application of TCR-Ts.

In contrast to CARs, TCRs have potential to recognize targets with any subcellular location. TCRs recognizing MHC-presented peptides derived from non-mutated tumor antigens, such as cancer testis or tissue-specific antigens that are found in many tumors of the same type, could be used to treat larger patient cohorts. However, isolation of TCRs targeting normal human proteins poses a major challenge because the TCR repertoire is pruned by thymic elimination of self-specific T cells^4^. To tap TCR repertoires capable of high-affinity binding to self-antigens complexed with self-HLA, different strategies are used to bypass thymic selection. Affinity maturation by introduction of selective mutations in the peptide-binding regions of TCRs is pursued to increase TCR affinity^5–9^. A consequence might be off-target reactivity since such TCRs have not undergone thymic selection^8,10^. Alternatively, T cells have been exposed to antigen in context of mismatched HLA. In this case the TCRs have been negatively selected on multiple HLA alleles, all of which have a high degree of structural similarity, except for the restricting HLA allele^11–15^. Yet another approach exploits that TCRs can react to human antigenic sequences that are absent in HLA-transgenic mice, where the T cells are negatively selected on the restricting HLA allele presenting the murine peptidome^16,17^. These strategies result in TCRs that may cause adverse effects when therapeutically applied unless carefully tested, because of varying risks for recognition of unintended targets. A risk may even apply to TCRs (whether directed against mutated or non-mutated tumor antigens) derived from thymus-selected human T cells that are used in a patient whose MHC composition differs from that of the donor. Answering this need, multiple studies have proposed strategies to map TCR-reactivity to select the optimal candidate TCR for clinical translation^5,18–23^. Although several of these technologies have provided important information to advance our understanding of TCR degeneracy, they are not easily applicable or accessible to most laboratories. Safety analyses of TCRs to date are therefore largely inconsistent and often incomplete, yet all preclinical studies should include a comprehensive screening for potential cross-reactivity.

**Fig. 1:**
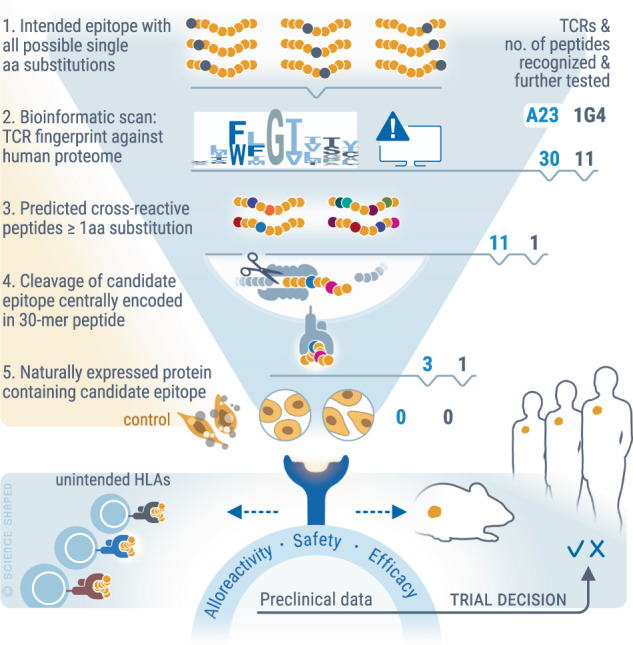
Schematic illustration of pipeline for preclinical testing of candidate therapeutic TCRs. Potential off-target reactivity of candidate therapeutic TCRs is first mapped using a peptide library containing the intended epitope with all possible single AA substitutions (Step 1). The TCR fingerprint is next screened against the human proteome using a computer algorithm. This bioinformatic search identifies potential cross-reactive epitopes containing one or any combination of multiple allowed AA substitutions (Step 2), which are then synthesized and tested for TCR-recognition in Step 3. Cleavage of candidate epitopes is studied by probing TCR-reactivity against target cells electroporated with mRNA constructs encoding 30-mer peptides containing the candidate cross-recognized epitope in the middle (Step 4). To evaluate if the candidate off-target epitope is processed and presented in a physiological setting, TCR-reactivity is probed against cell lines naturally expressing confirmed high levels of proteins containing the candidate off-target epitope (Step 5). The sum of preclinical data accumulated from (i) the off-target reactivity pipeline, (ii) assessment of potential recognition of unintended HLA-alleles and (iii) in vivo efficacy studies form the basis for an informed decision regarding clinical translation. Figure 1 is created by Ellen Tenstad, Science Shaped.

Here, we outline a series of experiments for a comprehensive safety testing of TCRs. The overview of our approach is summarized in Fig. 1. As benchmark for the in vitro pipeline, we relied on a clinically proven TCR; the HLA-A*02:01-restricted, affinity-matured NY-ESO-1-specific TCR 1G4-α95:LY^6^ (for brevity 1G4 TCR). This TCR is one of only few that have proven safe and efficacious in multiple completed, and on-going, trials^3,24,25^. In parallel, we tested the TCR A23 that is specific for an HLA-A*02:01-presented epitope derived from the clonal B-cell antigen CD20. We previously identified A23 from allo-restricted T cells, and testing of A23 TCR-Ts against a cell line panel suggested high HLA and peptide specificity^13,26^. TCR sequences are shown in Supplementary Fig. 1. We first determined TCR "fingerprints" of 1G4 and A23 to search for potentially cross-reactive peptides in the human proteome and then followed up candidates to determine if they represented off-targets in a physiological situation. By scanning against a panel of cell lines representing an extensive HLA-library, we then tested for cross-recognition of unintended HLA alleles. Isolation of TCRs from populations of allogeneic T cells carries the risk that TCR-Ts might recognize HLA-A*02:01 in complex with peptides other than the intended target. To further strengthen our in vitro analysis^13,26^, we tested the reactivity of A23 TCR-Ts in a syngeneic, transgenic HLA-A*02:01 mouse cancer model^27^, allowing us to evaluate the organs of successfully treated mice for T cell-induced autoimmunity.

## Results

### TCR “fingerprinting” to identify potential cross-reactivity to peptides in the human proteome

**Fig. 2:**
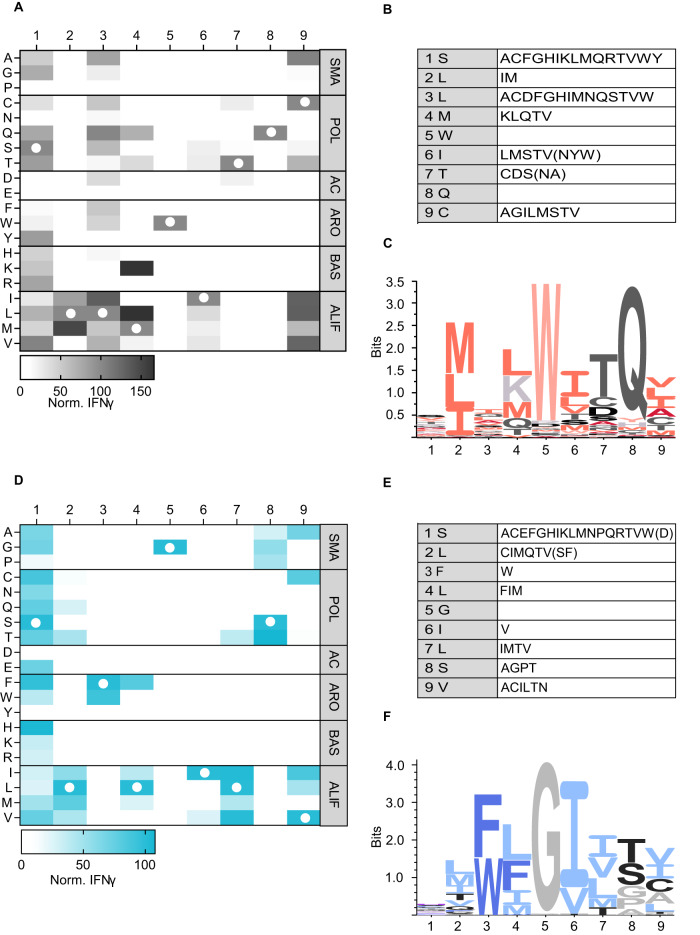
TCR fingerprinting of 1G4 and A23 using a positional scanning peptide matrix and a functional readout. 1G4 TCR-engineered T cells were incubated with HLA-A2^+^ B-LCL cells (**A**), and A23 TCR-Ts were incubated with HLA-A2^+^ K562 cells (**D**) loaded with a library of 9-mer peptides containing single amino acid exchanges compared to the cognate peptide. Supernatants of 24 h co-cultures were analyzed for IFN-γ content by ELISA. IFN-γ values are normalized to the response to the original peptide. Each heatmap shows the mean of three independent experiments with one technical replicate in each. Column/row intersections indicate the replaced amino acid at a given position, and white circles show the original peptide sequence. Substitutions are divided by physicochemical properties: SMA, small; POL, polar; AC, acidic; ARO, aromatic; BAS, basic; ALIF, aliphatic. **B**, **E** Overview of amino acid exchanges recognized at different AA positions that were used to query the curated human proteome databases UniProtKB/Swiss-Prot and Protein Data Bank by the ScanProsite tool when applying a cut-off of ≥10% or ≥5% (in parenthesis) of the IFN-γ production induced by the cognate peptide. Recognition pattern of the 1G4 TCR (**C**) and the A23 TCR (**F**), visualized as a sequence logo based on the data from (**A**, **D**).

First, we performed positional scanning of the 1G4 target peptide to identify possible cross-reactivities to peptides derived from proteins other than NY-ESO-1. 1G4 TCR-Ts (TCR sequence shown in Supplementary Fig. 1) were co-cultured with lymphoblastoid cells (LCLs, HLA-A*02:01^+^, NY-ESO-1^-^) loaded with the NY-ESO-1 peptide (SLLMWITQC, SLL) or derivative peptides containing single amino acid substitutions with all possible naturally occurring amino acids at each position, resulting in a matrix of 172 peptides, listed in Supplementary Table 1 with predicted binding affinities. Activation of 1G4 TCR-Ts was determined by measuring IFN-γ concentration in the culture supernatant (Fig. 2A). Lack of IFN-γ secretion indicated positions 2, 5, and 8 as crucial for TCR recognition because no exchanges, or only changes with structurally similar amino acids, were tolerated (Fig. 2A, B). Positions 1 and 3 were not essential for TCR recognition, while positions 4, 6, 7, and 9 were of intermediate importance. IFN-γ secretion and CD137 upregulation on activated TCR-expressing CD8^+^ T cells correlated strongly (gating strategy, results and correlation with IFN-γ for CD137 assay is shown in Supplementary Fig. 2A–C, *r* = 0.89). These data highly correlated with those found in another study^28^ (Supplementary Fig. 2D, *r* = 0.84). Activation of 1G4 TCR-Ts required the entire SLL peptide sequence, as nonamer peptides covering up- or downstream sequences, or longer and shorter SLL peptide variants, failed to induce IFN-γ secretion (Supplementary Fig. 2E). The resultant TCR fingerprint (Fig. 2C) was used to query curated proteome databases (UniProt, Swiss-Prot, Protein Data Bank), resulting in the identification of eleven potentially cross-reactive peptides, showing edit distances (number of minimal exchanges compared to the original sequence) between 4 and 7 (Supplementary Table 2). The list included predicted strong (*n* = 1) and weak binders (*n* = 3) to HLA-A*02:01.

To determine the recognition profile of A23 (TCR sequence shown in Supplementary Fig. 1), a similar strategy was applied using peptide-loaded K562 cells (HLA-A*02:01^+^, CD20^-^). The list of peptides with predicted binding affinities is shown in Supplementary Table 3. Lack of IFN-γ secretion identified the amino acids in position 3, 5, and 6 as most crucial for TCR-recognition, as no exchanges (position 5), or only substitutions with structurally similar amino acids (positions 3 and 6) were tolerated (Fig. 2D–F). Positions 2, 4, 7, 8, and 9 were more promiscuous, while position 1 could be exchanged with almost all other amino acids. Data for CD137-upregulation were strongly correlated (Supplementary Fig. 3A, B, *r* = 0.86). Activation was dependent on the SLF peptide, as nonamer peptides covering adjacent up- or downstream sequences of CD20, or truncated and longer SLF variants, could not induce full T-cell activation (Supplementary Fig. 3C). When querying the curated protein databases for peptides matching this recognition profile, 30 potentially cross-reactive peptides were identified (Supplementary Table 4). The edit distance varied between 2 and 7, with a predicted HLA-A*02:01 binding affinity that was lower for all as compared with the original SLF peptide (strong binders (*n* = 6), weak binders (*n* = 8), Supplementary Table 4).

### Investigating processing and presentation of off-target peptides

We next evaluated if peptides that were identified by 1G4 and A23 fingerprinting were cross-recognized by the respective TCR, and if so, whether they were naturally processed and presented on HLA-A*02:01^+^ human cells. The candidate off-target peptides (Supplementary Tables 2 and 4) were synthesized and loaded onto target cells that were co-incubated with the respective TCR-T.

**Fig. 3:**
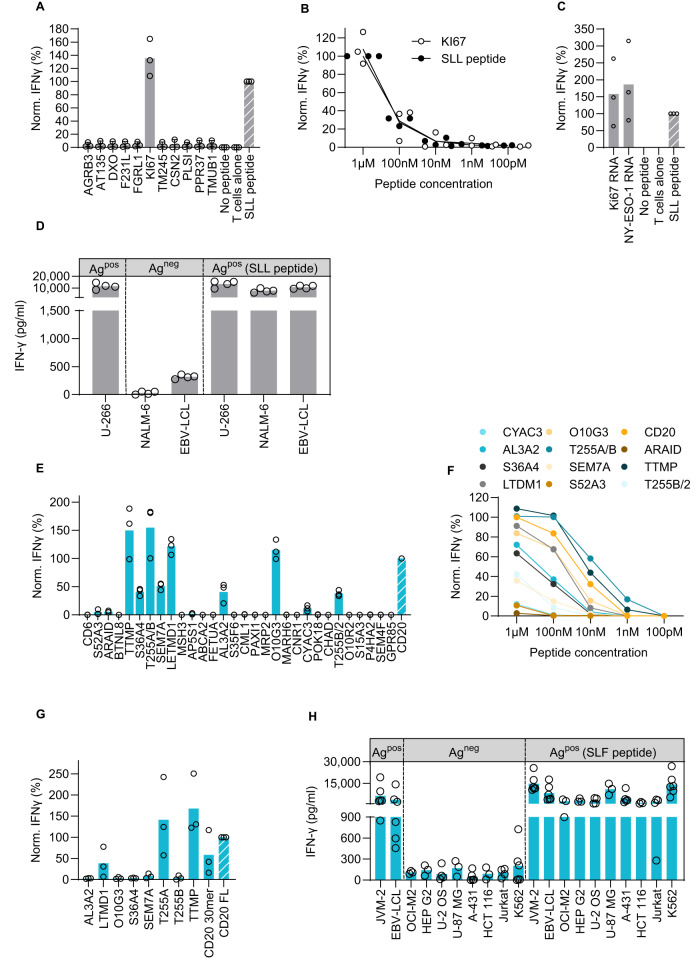
Mapping the off-target reactivity profile of 1G4 and A23 TCRs does not reveal clinically relevant cross-reactivities. 1G4 TCR-Ts were incubated with HLA-A2^+^ B-LCL cells that were either loaded with 9-mer peptides containing multiple amino acid exchanges compared to the original SLL peptide at a 10^−7^ M concentration (**A**), or at indicated concentrations (**B**), or electroporated with mRNA encoding 30-mer peptide sequences coupled to a GFP tag, containing potentially cross-recognized 9-mer peptides in the middle (**C**). Supernatants of 24 h co-cultures were analyzed for IFN-γ content by ELISA. The graphs show pooled data for three (**A**–**C**) or four (**D**) independent experiments with three technical replicates in each. Dots represent means of technical replicates. Values are normalized to the IFN-γ production induced by the original target peptide (range 2690–11,600 pg/ml). **D** HLA-A2^+^ cell lines expressing the intended target NY-ESO-1 (U-266), denoted Ag^pos^, or the potentially cross-reactive protein Ki67 (NALM-6, EBV-LCL) but not expressing NY-ESO-1 (Ag^neg^), were loaded or not with the NY-ESO-1 SLL peptide (100 nM). Cell lines were co-incubated with 1G4 TCR-Ts. After 24 h, T-cell activation was assessed by measuring the IFN-γ production by ELISA. The graph shows pooled data from 3 to 4 independent experiments each run with three technical replicates. Dots represent means of technical replicates. A23 TCR-Ts were incubated with HLA-A2^+^ K562 cells that were either loaded with 9-mer peptides containing multiple amino acid exchanges compared to the original SLF peptide at a 10^−7^ M concentration (**E**), or at indicated concentrations (**F**), or electroporated with mRNA encoding 30-mer peptide sequences coupled to a GFP tag, containing potentially cross-recognized 9-mer peptides in the middle (**G**). Supernatants of 24 h co-cultures were analyzed for IFN-γ content by ELISA. The graphs show pooled data for three independent experiments with three technical replicates in each. Dots represent means of technical replicates, except for (**F**), in which each dot denotes the mean of three independent experiments with three technical replicates in each. Values are normalized to the IFN-γ production induced by the original target peptide (range 5100–22,000 pg/ml). **H** HLA-A2^+^ cell lines expressing the intended target CD20 (JVM-2, EBV-LCL), denoted Ag^pos^, or the potentially cross-reactive proteins LETMD1 (OCI-M2, Hep-G2), TTMP (U-2 OS, U87-MG) or T255A (A-431, HCT-116) but not CD20, were loaded or not with the CD20 peptide SLF (100 nM). Cell lines were co-incubated with A23 TCR-Ts. After 24 h, T-cell activation was assessed by measuring the IFN-γ production by ELISA. The graph shows pooled data from 3 to 6 independent experiments with three technical replicates in each. Dots represent means of technical replicates.

Only one peptide, derived from Ki67, was able to induce IFN-γ secretion by 1G4 TCR-Ts (Fig. 3A). Titration revealed that 1G4 TCR-Ts recognized both the cognate peptide and the Ki67-derived peptide with very high and similar sensitivity (Fig. 3B). These data are consistent with a previously published study^28^. Natural processing and presentation of this Ki67-derived peptide on HLA-A*02:01 was, however, not investigated. To examine this further, we generated mRNA encoding the peptide of interest flanked by an additional 10 amino acids of the adjacent natural sequence and a GFP reporter at the end (mRNA 30-mer, Supplementary Fig. 4A). Ki67 and NY-ESO-1 peptides were properly expressed, processed and presented, as the mRNA constructs stimulated IFN-γ secretion in 1G4 TCR-Ts at similar levels (Fig. 3C). The predicted binding affinity of the Ki67 peptide to HLA-A*02:01 is even higher than that of the cognate peptide (6.1 nM vs. 666.7 nM, Supplementary Table 2). To test for endogenous presentation of the Ki67-derived peptide in human cells, we established a panel of HLA-A*02:01^+^ cell lines that naturally express high levels of Ki67 or NY-ESO-1. The cell lines were selected based on RNA sequencing data^29^, and expression was confirmed by qPCR on RNA level and flow cytometry or Western blot on protein level (Supplementary Fig. 5A and Supplementary Fig. 6A, B). 1G4 TCR-Ts secreted IFN-γ in response to a cell line endogenously expressing NY-ESO-1, and to NY-ESO-1-negative cell lines loaded with the SLL peptide. In contrast, cell lines endogenously expressing Ki67 (but not NY-ESO-1) were unable to induce activation (Fig. 3D), indicating that the Ki67-derived peptide is not naturally processed and presented from the native protein at levels sufficient to induce 1G4-mediated activation.

We next performed mass spectrometry-based immunopeptidomics to investigate if Ki67-derived peptides could be identified on HLA using mono-allelic B721.221 cell lines. Multiple Ki67 peptides were indeed identified on HLA-A (A*03:01, A*11:01) and B (B*40:01, B*44:02) alleles (Supplementary Fig. 7). However, no Ki67 peptides were found on HLA-A*02:01. Interestingly, while the FLTLWLTQV peptide was not detected on HLA-A*02:01^+^, peptides partly overlapping with this sequence were presented by both B*40:01 and B*44:02.

Eleven candidate off-target peptides activated the A23 TCR-Ts (Fig. 3E). Peptide titration identified eight that stimulated IFN-γ secretion also at physiologically more relevant, although still high, concentrations (Fig. 3F). These peptides had a higher predicted binding affinity to HLA-A*02:01 (*p* = 0.0502) and a significantly lower edit distance (*p* = 0.0210) than the non-activating peptides (Supplementary Fig. 3D). Processing and presentation were examined using mRNA constructs encoding 30-mers (Supplementary Fig. 4B). Three out of 8 mRNA constructs, encoding peptides from TTMP, LETMD1 and T255A, activated A23 TCR-Ts, indicating that these peptides were processed, presented and recognized by A23 (Fig. 3G). To check whether A23 TCR-Ts were activated by cells with native expression of the three genes, we assembled a set of cell lines showing high expression of either TTMP, LETMD1, T255A or CD20 (Fig. 3H). Expression of all four genes was verified by qPCR on RNA level (Supplementary Fig. 5B) and three on protein level (Supplementary Fig. 6C, D). Importantly, even though all genes encoding potentially cross-reactive peptides were expressed at high levels, none of the peptides could be detected by A23 TCR-Ts, as indicated by no or negligible IFN-γ production (Fig. 3H). A23 TCR-Ts recognized only cell lines that naturally expressed CD20 or were loaded with the SLF peptide. Thus, we concluded that these data did not provide evidence that A23 recognizes unintended peptides in cell lines with native protein expression levels.

### Screening TCR-Ts against a panel of lymphoblastoid cell lines representing an HLA class I library covering all major ethnicities

In addition to the potential risk of cross-reactivity of TCRs to unintended peptides, the possibility of TCRs reacting to HLA alleles other than the intended allele should be investigated to ensure safe application in ATTs. To this end, we assembled a panel of 33 LCLs (Supplementary Table 5) to cover the most frequently expressed HLA-A, B, and C alleles across multiple ethnic groups (allele frequencies are displayed in Supplementary Table 6).

**Fig. 4:**
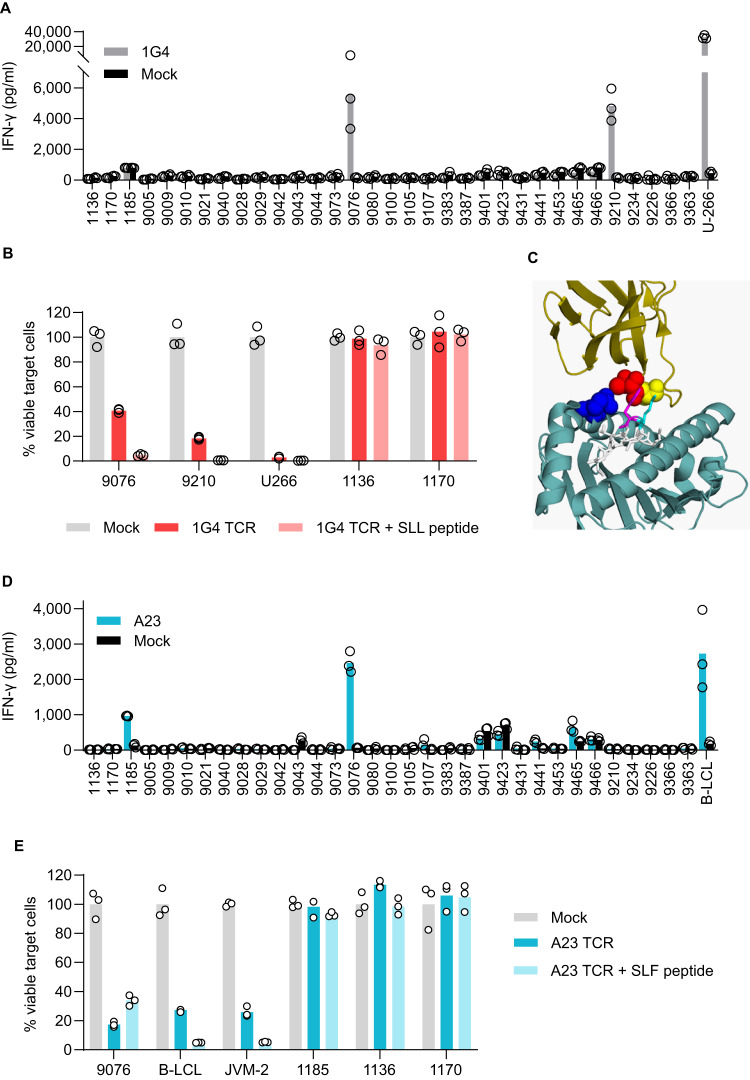
Mapping the allo-reactivity profiles of 1G4 and A23 TCRs reveals cross-recognition of unintended HLA-alleles within the HLA-A02 supertype. 1G4 TCR-Ts (**A**) and A23 TCR-Ts (**D**) were co-incubated with a panel of B-LCL cells with a known HLA type (listed in Supplementary Table 5). Supernatants collected after 24 h of co-culture were analyzed for IFN-γ content by ELISA. Bars shows mean from one experiment representative of two performed with dots representing technical replicates. Positive controls: U-266 A2^+^ (NY-ESO-1^pos^) in (**A**) and B-LCL A2^+^ (CD20 ^pos^) in (**D**). 1G4 TCR-Ts (**B**) or A23 TCR-Ts (**E**) were co- incubated with B-LCL lines expressing cross-reactive HLA alleles: For 1G4: 9076 (HLA-A*02:06- and HLA-A*02:07-positive) and 9210 (HLA-A*02:03-positive), for A23: 9076 (HLA-A*02:06- and HLA-A*02:07-positive) and 1185 (HLA-A*02:05-positive). After 48 h, number of surviving B-LCL cells was quantified by flow-cytometry and normalized to wells incubated with Mock cells. Loading with 100 nM of SLL or SLF peptide was included as a control. Positive controls: U266 (for 1G4) B-LCL and JVM2 (for A23). Negative controls (1136 and 1170). Figure shows one of two independent experiments performed with three technical replicates in each. Circles denote technical replicates. **C** Crystal structure of TCR 1G4 binding HLA-A*02:01 and NY-ESO-1_157_ (SLL peptide). 1G4 TCR: Ribbon model in dark yellow. Amino acids exchanged in mutant 1G4 are shown as spheres (Threonine (postion 95, red), Serine (position 96, yellow)). SLL peptide: Stick model in white. Amino acids that are in close spatial proximity to the TCRα chain of 1G4 are Methionine (position 4, cyan) and Tryptophan (position 5, magenta). HLA-A*02:01: Ribbon model in dark cyan. Conserved TCR contact residue that is in close spatial proximity to the TCRα chain of 1G4 is shown as sphere (Glutamine (position 155, blue)). Protein data bank file: 2BNR^32^. Visualized using PyMOL.

1G4 TCR-Ts secreted IFN-γ upon incubation with LCLs #9076 and #9210 suggesting cross-reactivities to HLA alleles A*02:03, A*02:06, and A*02:07 (A02 supertype^30^, Fig. 4A). Reactivity of 1G4 TCR-Ts against LCLs #9076 and #9210 was verified by assessing target cell killing (Fig. 4B). Since LCLs do not express NY-ESO-1^29^ (Supplementary Fig. 5A), irrelevant peptides in complex with these HLA alleles led to activation of 1G4 TCR-Ts in absence of the SLL peptide. Sequence alignment between the cross-reactive HLA alleles and HLA-A*02:01 showed that amino acids in HLA-A*02:01 that are involved in TCR binding^31^ are shared by all alleles (Supplementary Fig. 8). The importance of these conserved amino acids in HLA-A*02 for TCR binding may have implications for the reactivity of 1G4 TCR-Ts, as the α-chain of this affinity-enhanced TCR harbors mutations in critical positions that affect its reactivity^32^. A structural analysis shows that mutant position 95 in the TCRα chain of 1G4 is in close spatial proximity to the conserved amino acid 155, which is important for TCR binding^31^, possibly accountable for the cross-reactivity to these alleles that are otherwise structurally almost identical (Fig. 4C). Taken together, these data suggest that patients expressing these HLA alleles should be excluded from therapy with 1G4 TCR-Ts until this cross-reactivity has been explored further. While the frequency of the alleles identified in this HLA screening is low in people with European, Middle Eastern or North African ancestry (Supplementary Table 6), these alleles are more common in other ethnicities.

A23 TCR-Ts secreted IFN-γ upon incubation with LCLs #1185 and #9076, suggesting cross-reactivities to the HLA alleles A*02:05, A*02:06, and A*02:07, all belonging to the same HLA supertype as HLA*02:01^30^ (Fig. 4D). In cytotoxicity experiments, however, only the LCL line #9067 was killed (Fig. 4E), consistent with the lower IFN-γ production observed in response to LCLs #1185. LCLs are CD20^+^ and binding of the SLF peptide to the cross-reactive HLA alleles cannot be excluded (predicted binding affinity: A*02:05 (36.90 nM), A*02:06 (13.22 nM), and A*02:07 (2064.45 nM)). Thus, reactivity to either the SLF peptide bound to the respective HLA-A allele, or to the HLA-A allele combined with another, irrelevant peptide, cannot be distinguished. Until this matter has been sorted out, patients with HLA alleles A*02:05, A*02:06 and A*02:07 should therefore be excluded from potential future T cell therapies utilizing A23.

### A23 TCR-Ts rejecting established tumors in a syngeneic mouse model of cancer persist and show no off-target reactivities

**Fig. 5:**
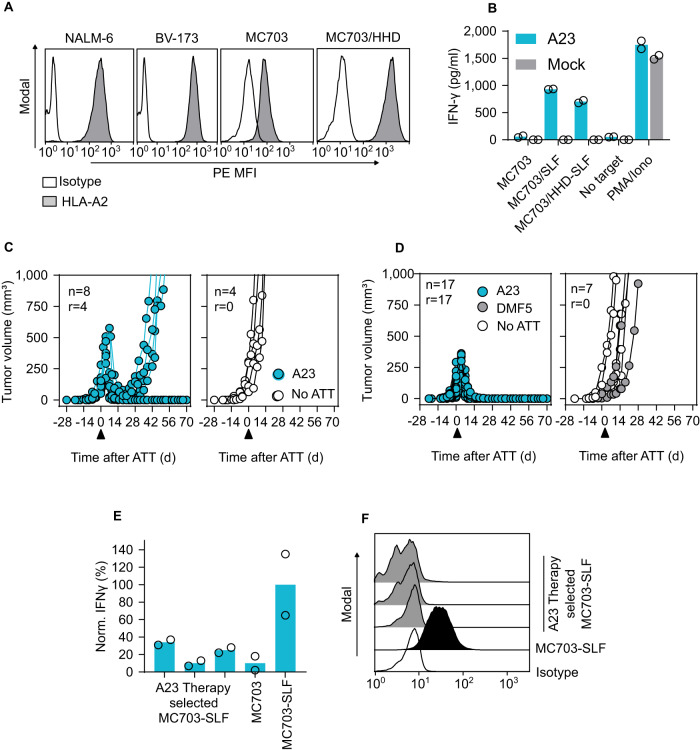
The CD20-specific A23 TCR mediates rejection of established tumors in a syngeneic mouse model of cancer. **A** Expression of HLA-A2 was measured by flow cytometry in the naturally HLA-A2^+^ human leukemia cell lines NALM-6 and BV-173 and in MC703 cells generated in HHD mice. To reach HLA-A2 levels in MC703 cells that are comparable to the human cell lines, the HHD transgene was retrovirally introduced (MC703/HHD). **B** MC703 and MC703-SLF tumor cells were incubated with A23 TCR-engineered HHD T cells and supernatants of 24 h co-cultures were analyzed for IFN-γ content by ELISA. One representative of two independent experiments is shown, and dots represent technical replicates. Growth of MC703-SLF (**C**) or MC703/HHD-SLF tumors (**D**) in HHDxRag1^−/−^ mice treated 3–4 weeks after tumor cell injection with A23 TCR-engineered HHD T cells (blue). Control animals received either HHD T cells engineered with TCR-DMF5 (MART-1 specific, gray) or were left untreated (no ATT, white). Numbers refer to treated animals (*n*) and to animals in which T-cell therapy achieved tumor rejection (*r*). The timepoint of adoptive T cell therapy (ATT) is indicated with an arrowhead. **E** MC703-SLF tumor cells either re-isolated from relapsed tumors depicted in (**D**) or cultured in vitro were incubated with A23 TCR-engineered HHD T cells. Supernatants of 24 h co-cultures were analyzed for IFN-γ content by ELISA. The relative amount of IFN-γ refers to the incubation of A23 TCR-Ts with MC703-SLF tumor cells cultured in vitro. Data shown are from one representative of two independent experiments performed, and dots represent technical replicates. **F** Expression of HHD on MC703-SLF tumor cells as determined by flow cytometry. Cells were derived from tumors that relapsed after treatment with A23 TCR-engineered HHD T cells depicted in (**D**) or from in vitro cultures.

To predict clinical activity of A23 TCR-Ts, we evaluated T-cell therapy in a syngeneic, HLA-A2-transgenic mouse cancer model^27^. Despite differences between human and mouse transcriptomes^33,34^, the syngeneic HLA-A*02:01-transgenic cancer model provides the opportunity to test for potential cross-reactivities to a multitude of HHD-presented peptides that are found on healthy murine tissues. Indeed, half of the 30 potentially cross-reactive human-derived peptides identified upon TCR fingerprinting were either sequence identical or only minimally different in mice (Supplementary Table 7). A23 was expressed in murine T cells isolated from HHD mice (Supplementary Fig. 9A) and incubation of TCR-Ts with titrated amounts of CD20 peptide recapitulated our results with human T cells^26^ (Supplementary Fig. 9B). HHDxRag1^−/−^ mice bearing CD20 peptide-expressing tumors (MC703-SLF, Fig. 5A, B) were treated with TCR-Ts when cancers were established (3–4 weeks after tumor cell injection) and tumors had an average size of 150 mm^3^. In this setup, A23 TCR-Ts achieved tumor rejection in half of the treated mice (Fig. 5C, left). The animals remained asymptomatic until the end of the experiment and showed no macroscopic signs of graft-versus-host disease. Untreated mice developed large tumors within 2–3 weeks and had to be sacrificed (Fig. 5C, right). MC703-SLF tumors that recurred after T-cell therapy (Fig. 5C, left) were poorly recognized by TCR-Ts in vitro (Fig. 5E), which can be attributed to the lack of measurable HHD surface expression in the re-isolated cancer cells (Fig. 5F).

Rejection of tumors in half of the treated mice indicated that the SLF-HHD complex was expressed at high enough levels to be recognized by the A23 TCR, and moreover that unintended peptide:HHD complexes were not recognized at levels causing adverse effects. Expression of the chimeric HLA-A*02:01 molecule HHD is, however, lower in PBMCs from HHD mice than expression of HLA-A*02:01 in human PBMCs^27^. Similarly, MC703 tumor cells derived from HHD mice showed lower HLA-A*02:01 expression than human leukemia cells (Fig. 5A, Supplementary Fig. 9C). To align MHC expression in MC703 tumor cells with the levels observed in human cancer, we additionally transfected the cells with the HHD transgene to increase surface levels (MC703/HHD, Fig. 5A, B, Supplementary Fig. 9C). We next treated MC703/HHD-SLF tumors grown in HHDxRag1^−/−^ mice, and A23 TCR-Ts were transferred when tumors had an approximate size of 100 mm^3^ and were established (3 weeks after tumor cell injection). Following T cell transfer, the tumor volume continued to increase for 4–5 days before the tissue collapsed and was rejected (Fig. 5D, left). All animals that were left untreated or that received unmodified T cells had to be sacrificed within 2–3 weeks after therapy start due to high tumor burden (Fig. 5D, right).

**Fig. 6:**
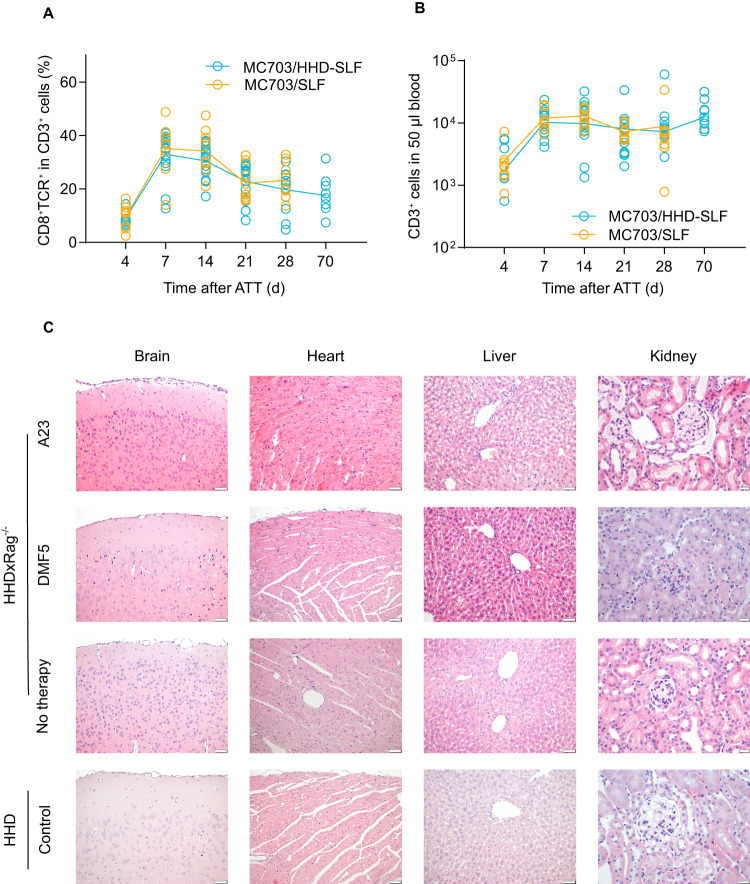
A23 TCR-Ts persist in treated animals and show no off-target reactivity in vivo to antigens presented on HHD. Peripheral blood samples from mice receiving T-cell therapy with A23 TCR-Ts were analyzed by flow cytometry. Graphs depict percentage of CD8^+^TCR^+^ HHD T cells within the CD3^+^ population (**A**) or total number of CD3^+^ HHD T cells (**B**) at indicated time points after ATT (adoptive T cell transfer) in mice bearing MC703-SLF (yellow) or MC703/HHD-SLF tumors (blue). **C** Indicated organs of HHDxRag1^−/−^ mice were resected 10 weeks after transfer of A23 TCR-Ts when tumors were rejected. Serial sections of organs were stained with hematoxylin and eosin. Representative pictures are shown. Organs from tumor-bearing HHDxRag1^−/−^ mice that were left untreated (5–7 weeks after tumor cell injection) or were treated with DMF5 T cells (4 weeks after therapy start) are shown as controls. Organs taken from untreated immunocompetent HHD mice are shown for comparison. Length of scale bar denotes 50 µm in brain, heart and liver sections and 20 µm in kidney sections.

A23 TCR-Ts showed peak expansion in peripheral blood of animals bearing MC703/HHD-SLF tumors on day 7 after therapy start and persisted in lower numbers also when tumors were rejected (Fig. 6A). The expansion of transferred CD3^+^ cells varied slightly between treated animals, yet the time course of T-cell expansion was consistent in all treated mice (Fig. 6B).

We selected animals for analysis that were observed for a period of 10 weeks after successful therapy (Fig. 5D). The animals remained asymptomatic until the end of the experiment and showed no macroscopic signs of graft-versus-host disease. Brain, heart, liver and kidney tissues were collected from HHDxRag1^−/−^ mice that were cured by treatment with A23 TCR-Ts. HHDxRag1^−/−^ mice that were either untreated or received TCR-Ts of unrelated specificity (targeting MART-1, DMF5), and immunocompetent HHD mice, were used as controls. Brain and heart tissue from mice receiving A23 TCR-Ts was normal. Liver tissue showed no additional changes relative to the reference samples, and only minor infiltration of macrophages and lymphocytes was observed (Fig. 6C). Minimal fibrous changes were found in the kidney tissue of control animals, which were accompanied by cellular infiltration of macrophages and lymphocytes in animals that received T-cell therapy (Fig. 6C). This pattern was, however, unrelated to the A23 TCR-Ts as it was also found in animals receiving DMF5 TCR-Ts.

## Discussion

Adoptive cell therapy with TCR-Ts offers an opportunity to target a wide range of malignant diseases^1–3,12,24^. Peptides derived from mutations, cancer-testis antigens and cell-type specific antigens can serve as targets in the context of various HLA molecules regardless of their cellular localization^6,12,35^. However, the inherent degeneracy of TCR recognition, combined with strategies that circumvent negative thymic selection to enrich for high-affinity TCRs, calls for a systematic and standardized pipeline for preclinical testing. The ultimate and challenging goal of such a pipeline is to avoid clinically unmanageable adverse events while ensuring efficacy, setting thresholds in vitro that correspond to tolerable off-target reactivities in different tissues. This will only be possible when detailed in vitro characteristics of many more TCRs can be correlated with clinical effects. Although parts of such analyses have already been suggested by others^21,36^, a practical validation of all required screening steps using clinically relevant TCRs was missing.

While probing TCR reactivity against cell line panels representing various tissues of the body provides important information^34^, it is impossible to screen for reactivity against every cell type in the body. Thus, peptide libraries in which each position in the cognate peptide are replaced by all other amino acids, one at a time (positional scanning peptide matrix^19,28,37^), can more comprehensively inform about TCR promiscuity. So-called TCR “fingerprinting” can be based on detected binding of pooled peptide–HLA multimers, mapping amino acid requirements for TCR-binding. Binding of a particular pHLA-complex does, however, not necessarily lead to T-cell activation^38,39^. In contrast, a functional readout in response to antigen-presenting cells loaded with individual peptides directly identifies amino acid substitutions capable of activating TCR-Ts.

Once the permitted amino acid replacements are mapped, candidate cross-reactive peptides in the human proteome can be found in a bioinformatic search. For the TdT-specific TCRs T1 and T3^12^, no such candidates were identified, whereas for 1G4 and A23, the algorithm identified 11 and 30 such peptides with up to 7/9 replacements. The second peptide library thus provides the possibility to detect reactivity to peptides that have little homology with the cognate peptide. Predicted binding affinities ranged from high to low. The top candidate HLA-A*02:01-binders FLTLWLTQV (Ki67) and WLFFGITGL (CD6) were predicted to bind with high affinities. Remarkably, the Ki67 peptide induced almost identical activation of 1G4 TCR-Ts as the cognate peptide. The CD6 peptide, on the other hand, induced no reactivity in A23 TCR-Ts, demonstrating that prediction of MHC-binding alone cannot correctly identify cross-reactive peptides of clinical relevance.

Among the A23 off-target candidates, the median edit distance of TCR-activating peptides (four) was significantly lower than for peptides not causing TCR activation (five) (Supplementary Fig. 3D), indicating that higher sequence homology correlates with higher risk of TCR cross-reactivity. These results are in concordance with other assays showing that peptides that are cross-recognized by HLA-class I-restricted TCRs have a minimum of 3–4 positions that are conserved relative to the “original” peptide, often with additional structural similarities (such as the demand for hydrophobic amino acids in certain positions^20,22,23^).

The expression of short mRNAs encoding 30-mer peptides, with the cross-reactive peptide in the middle and its natural sequence flanking it in the genome, showed that only four out of 12 peptides were processed and presented on HLA-A*02:01. Among them was the mRNA encoding the FLTLWLTQV peptide of Ki67, which activated 1G4 TCR-T with similar efficacy as the 30-mer containing the SLLMWITQC peptide of NY-ESO-1. Considering that Ki67 is a nuclear protein associated with proliferation^40^, one would expect severe side effects from 1G4 T cell-therapy due to recognition of Ki67-expressing cells. However, despite efficient cleavage and presentation of the peptide from the 30-mer, target cells naturally expressing confirmed high levels of full-length Ki67 were not recognized by 1G4, consistent with the observed safe clinical profile. Additionally, we did not detect the FLTLWLTQV peptide of Ki67 in immunopeptidomics analysis of HLA-eluted ligands from monoallelic HLA-A*02:01^+^ B7.21.221 cell lines, while multiple other Ki67-derived peptides presented on different HLA alleles were identified. One possible explanation for the discrepancy in recognition between Ki67 mRNA-electroporated target cells and cells naturally expressing Ki67 could be that proteasomal cleavage in cells loaded with mRNA may be affected by the high amounts of Ki67 protein. While electroporation with Ki67 mRNA is sufficient for determining whether the epitope can be generated, overloading the cellular degradation machinery may lead to incomplete digestion of the Ki67 protein, thereby concealing the fact that the target epitope is destroyed when expressed at lower, natural levels. Indeed, the Ki67 epitope FLTLWLTQV that is recognized by 1G4 TCR-Ts shows 3 potential cleavage sites for which proteasomal activity is predicted with high probability (positions 4 (97.53%), 5 (87.42%), and 6 (66.34%), https://services.healthtech.dtu.dk/services/NetChop-3.1/, 05/12/23). Similar results were observed for the three epitopes (TTMP – SIFLGVITV, LETMD1 – CLFLGIISI, and T255A GLFLGIITA) cross-reactive with the A23 TCR. Target cells transfected with these mRNAs strongly activated A23 TCR-Ts, yet target cells expressing the corresponding full-length versions of these ubiquitously expressed proteins did not induce IFN-γ production. These results demonstrate the importance of investigating TCR-T reactivity to cells with native expression for detection of clinically relevant cross-reactive epitopes. Screening of TCR-T reactivity to target cells equipped with genetically encoded (DNA or mRNA), overexpressed polypeptides^22^ therefore require downstream validation, as valuable TCRs otherwise might be disregarded. However, it is worth noting that the identification of cross-reactive peptides based on TCR fingerprinting does not exclude the existence of epitopes that are structurally divergent from the cognate epitope but still recognized by the TCR.

To investigate potential TCR cross-recognition of peptides presented on HLA class I alleles other than HLA-A*02:01, we assembled a panel of 33 lymphoblastoid cell lines^18,19^ with known HLA types (Tables S5 and S6) covering the majority of HLA alleles across multiple ethnic groups. None of the B-LCLs expressing HLA alleles outside of the A02 supertype^30^ activated 1G4 or A23 TCR-Ts, indicating lack of reactivity to structurally different HLA alleles. Our results showed, however, that 1G4 TCR-Ts reacted to B-LCLs expressing A*02:03, A*02:06, and A*02:07. As B-LCLs do not express NY-ESO-1, this indicates that 1G4 recognizes at least one other peptide in context of these alleles. The allele frequency of the cross-reactive alleles identified in our HLA screening is low in people with European, Middle Eastern or North African ancestry (Supplementary Table 6), but higher in other ethnicities (Supplementary Table 6). A23 TCR-Ts reacted to B-LCLs expressing A*02:05, A*02:06 and A*02:07. Since B-LCLs also express CD20, potential recognition of the cognate SLF-CD20 peptide presented on other HLA alleles cannot be distinguished from potential cross-reactivity to other peptides. Our results emphasize the importance of testing for TCR-reactivity against a panel of cell lines expressing a diverse repertoire of HLA alleles, with consequences for patient selection in clinical trials. Since the 1G4 TCR has lost its dependence on the CD8 coreceptor due to affinity maturation^6^, this cross-reactivity has implications for TCR-engineered CD8 and CD4 T cells when used for T cell therapy.

To predict clinical activity of the A23 TCR-Ts, we made use of an HLA-A*02:01-transgenic syngeneic mouse cancer model^27^. We showed that A23 TCR-Ts eradicated established tumors (Fig. 5D) and were detectable in peripheral blood throughout the 70 day observation period (Fig. 6A, B). This syngeneic model is not limited by the confounding effects introduced when analyzing TCR-Ts in common xenograft models, where adoptively transferred human T cells may attack tumor cells when derived from other donors (allo-reactivity) or attack normal cells of the murine host (xeno-reactivity). Moreover, interaction between TCR-Ts and tumor stroma cells are permitted, which allows analysis close to the situation in the patient. We did not observe any autoimmune side effects caused by the T cells in murine tissues, expressing the restricting HLA-A*02:01, supporting lack of off-target reactivity (Fig. 6C). The comparison between human and corresponding mouse sequences of investigated potential off-target epitopes of A23 is shown in Supplementary Table 7. However, due to inter-species differences between the human and murine proteome, proteasomal cleavage, and the TAP transporter^41^, this does not prove lack of off-target reactivity in humans. A limitation of the syngeneic model is the need for ectopic expression of target epitopes, which does not reflect physiological levels of target epitopes and HLA of patient tumor cells.

The in vitro pipeline presented here follows the systematic steps (outlined in Fig. 1) of 1) TCR fingerprinting using a functional readout, 2) bioinformatic identification of candidate cross-recognized peptides, 3) candidate validation, followed by 4) validation of cleavage sites using short mRNA constructs and 5) validation of endogenous presentation from natively expressed proteins, and finally 6) screening for reactivity against non-intended HLA alleles. The use of peptide libraries at high concentrations allows for detection of candidate cross-reactive peptides with high sensitivity. The approach involves multiple steps but can readily be performed by a single staff member. It is based on commonly used techniques and commercially available reagents and can easily be adapted to any TCR and its target, facilitating wide-spread use in laboratories developing TCRs for clinical applications. The pipeline was validated with the clinically successful 1G4 TCR, showing results consistent with lack of severe side effects reported in patients. However, previously undescribed cross-reactivity to three unintended HLA alleles suggests that patients expressing these alleles should not be treated with 1G4 TCR-Ts. Testing of A23 TCR-Ts also suggested a favorable safety profile, as well as a high degree of efficacy in vivo. Accumulation of corresponding data for other therapeutic TCRs could facilitate future determination of cut-off values for in vitro reactivity that correspond to clinically manageable toxicities. While no T-cell reactivity is acceptable to non-replaceable tissues essential for survival, further studies could examine the possibility whether some degree of on-target toxicity to well-regenerating tissues can be tolerated to increase the number of patients eligible for therapy, especially in combination with TCR products coupled with off-switches or suicide genes^42^.

## Methods

### Cells

The following cell lines were obtained from American Type Culture Collection and German Collection of Microorganisms and Cell Cultures: K562 (CCL-243), JVM-2 (CRL-3002), OCI-M2 (ACC 619), HEP G2 (ACC 180), U-2 OS (HTB-96), U-87 MG (HTB-14), A-431 (CRL-1555), HCT 116 (CCL-247), Jurkat (TIB-152), NALM-6 (CRL-3273), Phoenix-Ampho (CRL-3213), U266 (ACC 9). Cell lines were cultured as instructed by the manufacturers, were regularly tested for mycoplasma contamination (MycoAlert PLUS Mycoplasma Detection Kit, LT07-318) and for authenticity and purity by STR analysis (LabCorp).

HLA-A*02:01 negative cell lines were retrovirally transduced to express HLA-A*02:01. HLA-typed lymphoblastoid cell lines (LCLs) covering the most frequent HLA alleles across multiple ethnicities (Supplementary Table 3) were used to determine potential cross-recognition of multiple HLA alleles, and B721.221 cells retrovirally transduced to express single HLA were used in mass spectrometry (MS) experiments (Fred Hutchinson Cancer Center and Merck). Allele frequencies are displayed in Supplementary Table 6, which were used to calculate the population prevalence using the Hardy–Weinberg principle with the IEDB analysis resource (http://tools.iedb.org/population/). The alignment of the sequences of HLA-A*02:01, -A*02:03, -A*02:06, and -A*02:07 in shown on Supplementary Fig. S8. Plat-E cells^43^ were used for generation of retroviral supernatants for transduction of murine cells. MC703 cells^27^ were transduced with MP71-SLF-mCherry and MP71-HHD-i-GFP. MC703-SLF tumor cell lines were generated by transducing 5 × 10^4^ MC703 cells with MP71-SLF-mCherry and MP71-HHD-i-GFP twice. Bulk cultures were expanded and enriched by preparative FACS using GFP and/or mCherry as marker (MC703/HHD, MC703-SLF, MC703/HHD-SLF)^44^.

### Retroviral and RNA constructs

cDNA sequences encoding A23, 1G4 and DMF5 TCRs and HLA-A*02:01 (reported in earlier publications^6,26,45^) were synthesized by Genscript and cloned into a pMP71 vector. The transgene cassettes of all TCRs contain mouse constant regions with additional cysteine residues to limit mispairing. A porcine teschovirus–derived 2A sequence (P2A) was used to link the TCR genes in a β-P2A-α configuration. The TCR transgene cassettes were codon optimized to maximize TCR expression and pairing. TCR sequences shown in Supplementary Fig. 1. RNA constructs were prepared encoding 30 AA long sections containing a potentially cross-reactive 9 AA long peptide in the middle, with a green fluorescent protein (GFP) tag to control for transfection efficiency (Supplementary Fig. 4). A fusion construct of a trimer minigene (SLFLGILSV-AAY (SLF)) and mCherry was integrated into pMP71-PRE (pMP71-SLF-mCherry)^26^. The gene encoding HHD^46^ was ligated into pMP71-i-GFP^47^ via Eco72I restriction site to generate the retroviral vector plasmid pMP71-HHD-i-GFP.

### Peptides and positional scanning

Peptides were synthesized by GenScript to >70% purity, dissolved in DMSO (10 mg/ml), stored at −80 °C. Before functional tests, target cells were peptide-loaded for 2 hs at indicated concentrations. Positional scanning of TCR target peptides was performed as described in detail in the results section. Predicted binding affinities of all peptide mimotopes used in our study are shown in Supplementary Tables 1 and 3. A peptide that induced IFN-γ production by TCR-Ts (in ELISA, see below) corresponding to 10% or more of the response to the cognate peptide, was considered positive/cross-recognized (Fig. 2B, E). Additional peptides inducing responses between 5 and 10% of the original peptide were also included (Fig. 2B, E, in parenthesis). All possible combinations of “tolerated” AA exchanges were queried against the curated human proteome databases UniProtKB/Swiss-Prot and Protein Data Bank using the ScanProsite tool to identify natural peptides to which the TCRs would have potential cross-reactivity. The lists of identified peptides are shown in Supplementary Tables 2 and 4. The comparison of human and corresponding mouse sequences of investigated potential off-target epitopes of A23 is shown in Supplementary Table 7.

### T-cell activation and killing assays

5 × 10^4^ human or murine TCR-Ts were co-incubated (E:T ratio 1:2 (human) 1:1 (murine)) for 24 h with target cells. TCR-Ts were not selected for CD8. Target cells were loaded with peptide for 2 h, or electroporated with mRNA at a 100 µg/ml concentration. Functionality of the TCR-Ts was assessed by measuring surface CD137 up-regulation of live/CD8+/mouse TCRβ+ cells (flow cytometry, gating strategy shown in Supplementary Fig. 2A) and IFN-γ release (ELISA). For TCR-independent maximal stimulation, 1 µM ionomycin (Merck, I3909-1ML) and 5 ng/ml phorbol-12-myristate-13-acetate (Merck, P1585-1MG) were used.

To quantify the IFN-γ production by T cells, Human or Mouse IFN-γ ELISA Sets (OptEIA, BD Biosciences, 555138 and 555142) were utilized following the protocol provided by the vendor. Data are reported in pg/ml either as a single experiment with technical replicates or as the mean of three independent experiments. In the latter case values are normalized to the IFN- γ production induced by the original target peptide.

To detect T-cell numbers in peripheral blood, 50 µl peripheral blood was incubated with Fc block (BD, 14-9161-73 and BioLegend, 101320) and indicated antibodies. Total cells in each sample were measured to determine total cell counts per 50 µl blood. Numbers of CD3^+^ cells were calculated per ml blood. Gating strategy is shown in Supplementary Fig. 9D.

For cytotoxicity assays, 1G4 or A23 TCR-T cells were CTV (cell trace violet) labeled and co-cultured with cell lines expressing or lacking the cross-reactive alleles in a 2:1 effector: target ratio, in triplicates. As an additional control, loading with the original target peptide was performed. Only TCR transduced PBMCs with >80% TCRβ^+^ of live CD8^+^ cells were used. After 48 h, remaining cells were harvested and stained for flow cytometry, including the addition of CountBright Absolute Counting Beads (Thermo Fisher) at the final step. An equal number of bead events in each well were acquired on LSR II flow cytometer (BD Biosciences).

### Mice, tumor challenge, and adoptive T-cell transfer

The HHD molecule is a fusion of the α1 and α2 domains of HLA-A*02:01 that form the peptide binding groove, fused to the α3 domain of H-2D^b^ which anchors the molecule in the cell membrane and provides the binding site for mouse CD8. Human β_2_-microglobulin is fused to the α1 domain with a separating GSG linker element. To achieve higher surface expression of the chimeric MHC construct, HHD mice lack expression of endogenous H-2^b^ alleles^48^. HHD mice were provided by François A. Lemonnier (Institute Pasteur, Paris, France), generation of HHDxRag1^−/−^ mice has been described^27^. 3–5 × 10^6^ MC703-SLF or MC703/HHD-SLF cells were subcutaneously injected in 100 µl PBS into the left flank of HHDxRag1^−/−^ mice (12–20 weeks old, female or male). Tumor growth was analyzed 2–3 times a week by determination of tumor volume using caliper measurements according to π/6 × (abc). Mice were randomized into experimental groups so that mean tumor size was comparable among groups. On the day of T-cell transfer, mice were ranked by tumor size and sequentially allocated to treatment groups to ensure equal average tumor sizes between groups. Mice were treated with HHD TCR-Ts earliest 3–4 weeks after tumor cell injection. HHD TCR-Ts were analyzed for expression of CD8, A23 (TCRvβ17), and DMF5 (A2/K^b^:ELA) by flow cytometry and intravenously injected in 100 µl PBS (adjusted to 1 × 10^6^ CD8^+^TCR^+^ HHD T cells per mouse) 3 days after transduction. Examiners were not blinded to treatment groups. Mice were sacrificed when the tumors reached the maximum permitted size or if the overall health-condition of animals was poor due to tumor burden. Animals were excluded from analysis if they died due to reasons unrelated to tumor burden or T-cell therapy.

### Antibodies and flow cytometry

Flow cytometry was performed on a BD LSR II, MACSQuant or FACSCalibur flow cytometers (BD Biosciences) and data was analyzed using FlowJo (TreeStar) software. Cells were treated with Human Fc Receptor binding inhibitor (eBioscience) or anti-mouse CD16/CD32 (2.4G2, BioLegend, Cat. No 101302, dilution 1:100) followed by the addition of antibodies for 15 min on ice. When necessary, erythrocytes were lysed by ammonium chloride treatment. The following fluorescently labeled antibodies were used: anti-human - CD8a (FITC or Brilliant Violet 421, RPA-T8, BioLegend, Cat. No 100706 and 100738, 1:100 and 1:150), anti-human - CD137 (Alexa Fluor 647, 4B4-1, Thermo Fisher Scientific, Cat. No A51019, 1:200), anti-human - HLA-A:02*01 (PE or Alexa Fluor 647, BB7.2, BioLegend and BioRad, Cat. No 343306 and MCA2090A647, 1:100 and 1:30) anti-mouse TCRβ chain (PE, H57-597, BD Biosciences, Cat. No 553173, 1:100), anti-mouse - CD3 (APC, 145-2C11, BioLegend, Cat. No 100312, 1: 100), anti-mouse - CD8 (Brilliant Violet 421, 53-6.7, BioLegend, Cat. No 100738, 1:200) and isotype control antibody (mouse IgG2b, Alexa Fluor 647, BioRad, Cat. No MCA691, 1:30). In some cases, anti-human TCRvβ-specific antibodies (A23: TCRvβ17 clone E17.5F3.15.13 PE from Beckman Coulter, Cat. No IM2048, 1:30) or chimeric A2/Kb pentamers (DMF5 TCR) loaded with ELAGIGILTV peptide (PE, ProImmune, custom produced, 1:30) were used to analyze the expression of TCRs. In some experiments, Cell Trace Violet (CTV, Thermo Fisher Scientific, Cat. No C34557, 1:2000) labeling was used to distinguish between target and effector cells. Live/Dead Fixable Near-IR Dead Cell Stain kit or SYTOX Blue (both Thermo Fisher Scientific, Cat. No L34976 and S34857, 1:1000) were used to exclude dead cells in flow cytometry experiments.

### Pathological analysis

Indicated specimens were fixed in 10% formalin, embedded in paraffin, stained with haematoxylin and eosin, and evaluated by a pathologist in a blinded manner.

### Softwares and statistical analysis

Statistical analysis was performed by GraphPad Prism, version 8 (RRID: SCR_002798). Sequence logos were produced by Seq2Logo^49^ (RRID: SCR_008520). Results of mimotope screens were probed against the curated human proteome databases UniProtKB/Swiss-Prot and Protein Data Bank using the ScanProsite tool^50^ to identify potentially cross-reactive proteins. HLA-A*02:01 binding affinity and Rank was predicted by NetMHC 4.0. Peptides with %rank<0.5 or %rank<2 were considered strong and weak binders.

### Study approvals

This study was approved by the Regional Committee for Medical and Health Research Ethics (REC) South-East, Norway (no. 2018/879), the Institutional Review Board and the Data Protection Officer, Oslo University Hospital and was performed in accordance with the Declaration of Helsinki. Written informed consent was obtained from healthy blood donors prior to participating in the study. Animal experiments were approved by the German State Office for Health and Social Affairs (Landesamt für Gesundheit und Soziales). All experiments were performed in compliance with the institutional guidelines and 2010/63/EU directive on the protection of animals used for scientific purposes.

### Reporting summary

Further information on research design is available in the Nature Research Reporting Summary linked to this article.

### Supplementary information

Supplementary Material

Reporting Summary

## Data Availability

The data that support the findings of this study are included in the manuscript and in the Supplementary information. Additional datasets used in the study are: curated human proteome databases UniProtKB/Swiss-Prot and PDB by ScanProsite tool (https://prosite.expasy.org/scanprosite/); and peptide–MHC class I binding prediction algorithm NetMHC v.4.0 (http://www.cbs.dtu.dk/services/NetMHC/). The datasets generated during the current study are available from the corresponding author upon reasonable request.
