# Supplementary material for: A systematic safety pipeline for selection of T-cell receptors to enter clinical use
Source: NPJ Vaccines. 2023 Aug 22;8:126. doi: 10.1038/s41541-023-00713-y (PMC10444760; doi:10.1038/s41541-023-00713-y)
Supplement: Supplementary file 1 — Supplementary Material [file 41541_2023_713_MOESM1_ESM.pdf]

Supplementary Figure 1

Leader sequence
CDR3s
Murine constant domain with Cysteine substitution C
ribosomal skipping sequence P2A

1G4 TCR aa sequence

MSIGLLCCAALSLLWAGPVNAGVTQTPKFQVLKTGQSMTLQCAQDMNHEYMSWYRQDPGMGLRLIHYSVGAG
ITDQGEVPNGYNVSRSTTEDFPLRLLSAAPSQTSVYF**CASSYVGNTGELFF**GEGSRLTVL**EDLRNVTPPKVSLFEPSK**
AEIANKQKATLVCLARGFFPDHVELSWWVNGKEVHSGV CTDPQAYKESNYSYCLSSRLRVSATFWHNPRNHFR
CQVQFHGLSEEDKWPEGSPKPVTQNISAEAWGRADCGITSASYHQGVLSATILYEILLGKATLYAVLVSGLVLMA
MVKKKNS*GSGATNFSLLKQAGDVEENPGP*MKSLRVLLVILWLQLSWVWSQ KQEVTQIPAALSVPEGENLVLNCS
FTDSAIYNLQWFRQDPGKGLTSLLLIQSSQREQTSGRLNASLDKSSGRSTLYIAASQPGDSATYL **CAVRPLYGGSYIP**
TFGRGTSLIVHP**DIQNPEPAVYQLKDPRSQDSTLCLFTDFDSQINVPKTMESGTFITDKCVLDMKAMDSKSNGAI**
AWSNQTSFTCQDIFKETNATYPSSDVPCDATLTEKSFETDMNLNFQNLSVMGLRILLLKVAGFNLLMTLRLWSS

A23 TCR aa sequence

MSNQVLCCVVLCLLGANTVDGGITQSPKYLFRKEGQNVTLSCEQNLNHDAMYWYRQDPGQGLRLIYYSQIVNDF
QKGDIAEGYSVSREKKESFPLTV TSAQKNPTAFYL**CATAPGLSYEQYF**GPGTRLTVT**EDLRNVTPPKVSLFEPSKAEI**
ANKQKATLVCLARGFFPDHVELSWWVNGKEVHSGV CTDPQAYKESNYSYCLSSRLRVSATFWHNPRNHFRCQ
VQFHGLSEEDKWPEGSPKPVTQNISAEAWGRADCGITSASYHQGVLSATILYEILLGKATLYAVLVSGLVLMAM
VKKKNS*GSGATNFSLLKQAGDVEENPGP*MKSLRVLLVILWLQLSWVWSQ QQPVQSPQAVILREGEDAVINCSSSK
ALYSVHWYRQKHGEAPVFLMILLKGGEQKGHDKI SASFNEKKQQSSLYLTASQLSYSGTYF **CGTQGGSEKLVF**GKG
TKLTVNP **DIQNPEPAVYQLKDPRSQDSTLCLFTDFDSQINVPKTMESGTFITDKCVLDMKAMDSKSNGAIAWSN**
QTSFTCQDIFKETNATYPSSDVPCDATLTEKSFETDMNLNFQNLSVMGLRILLLKVAGFNLLMTLRLWSS

Supplementary Figure 1. Amino Acid sequences of the 1G4 and A23 TCRs

Colour coding denotes Leader sequence, CDR3s, Murine constant domain with Cysteine substitution C, and ribosomal skipping sequence P2A

Supplementary Figure 2

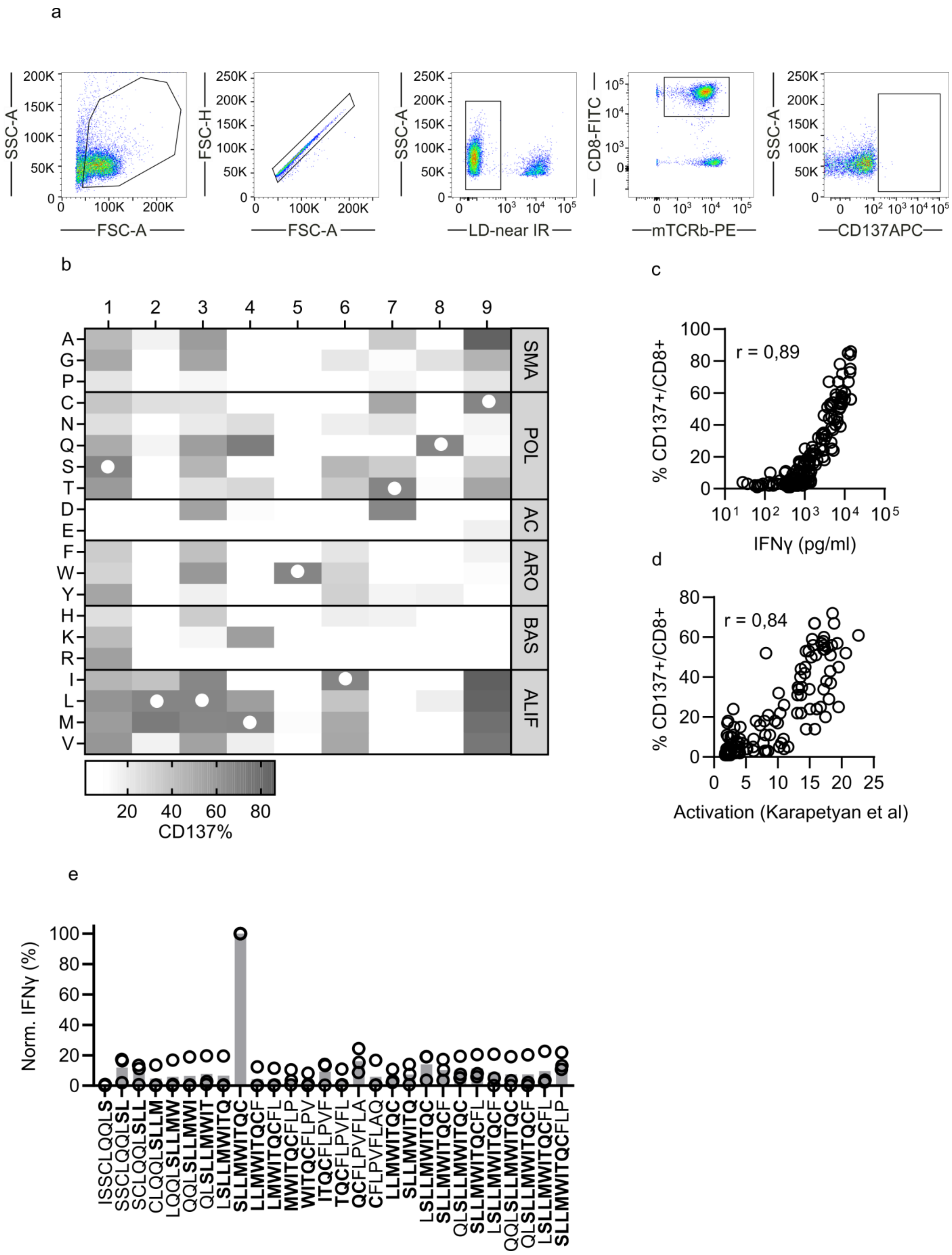

Supplementary Figure 2. 1G4 TCR-T activation in response to positional scanning peptide matrix and length variants of the cognate peptide

(A) Gating strategy for assessment of the percentage of transduced $CD8^+$ T cells expressing the activation marker CD137. Effector cells were identified as live single cells staining positively with CD8 and anti-mouse TCRβ.

(B) 1G4 TCR-Ts were incubated with $HLA\text{-}A2^+$ B-LCL cells loaded with a library of 9-mer peptides containing single amino acid exchanges compared to the original SLL peptide. Cells from 24 h co-cultures were analysed for CD137 upregulation by flow cytometry. The heat map shows the mean of three independent experiments. Column/row intersections indicate the replaced amino acid at a given position and white circles indicate the original peptide sequence. Substitutions are divided by physicochemical properties: SMA: small; POL: polar; AC: acidic; ARO: aromatic; BAS: basic; ALIF: aliphatic.

(C) Correlation analysis of the percentage of cells expressing CD137 (shown in Supplementary Figure 1A) with IFN-γ release (shown in Figure 1A). r = Pearson correlation coefficient.

(D) Correlation analysis of the percentage of cells expressing CD137 (shown in Supplementary Figure 1A) with activation marker upregulation published by Karapetyan et al.[28]. r = Pearson correlation coefficient.

(E) 1G4 TCR-Ts were incubated with $HLA\text{-}A2^+$ B-LCL cells loaded with peptides that are upstream or downstream of the original epitope, or longer peptides containing the SLF sequence at a 10^{-7} M concentration. Supernatants of 24 h co-cultures were analyzed for IFN-γ content by ELISA. The graphs show pooled data from 3 independent experiments with three technical replicates in each. Dots represent means of technical replicates. Values are normalized to IFN- γ production induced by the original target peptide (range 9,900 – 12,500pg/ml).

CDR3s, Murine constant domain with Cysteine substitution C, and ribosomal skipping sequence P2A

Supplementary Figure 3

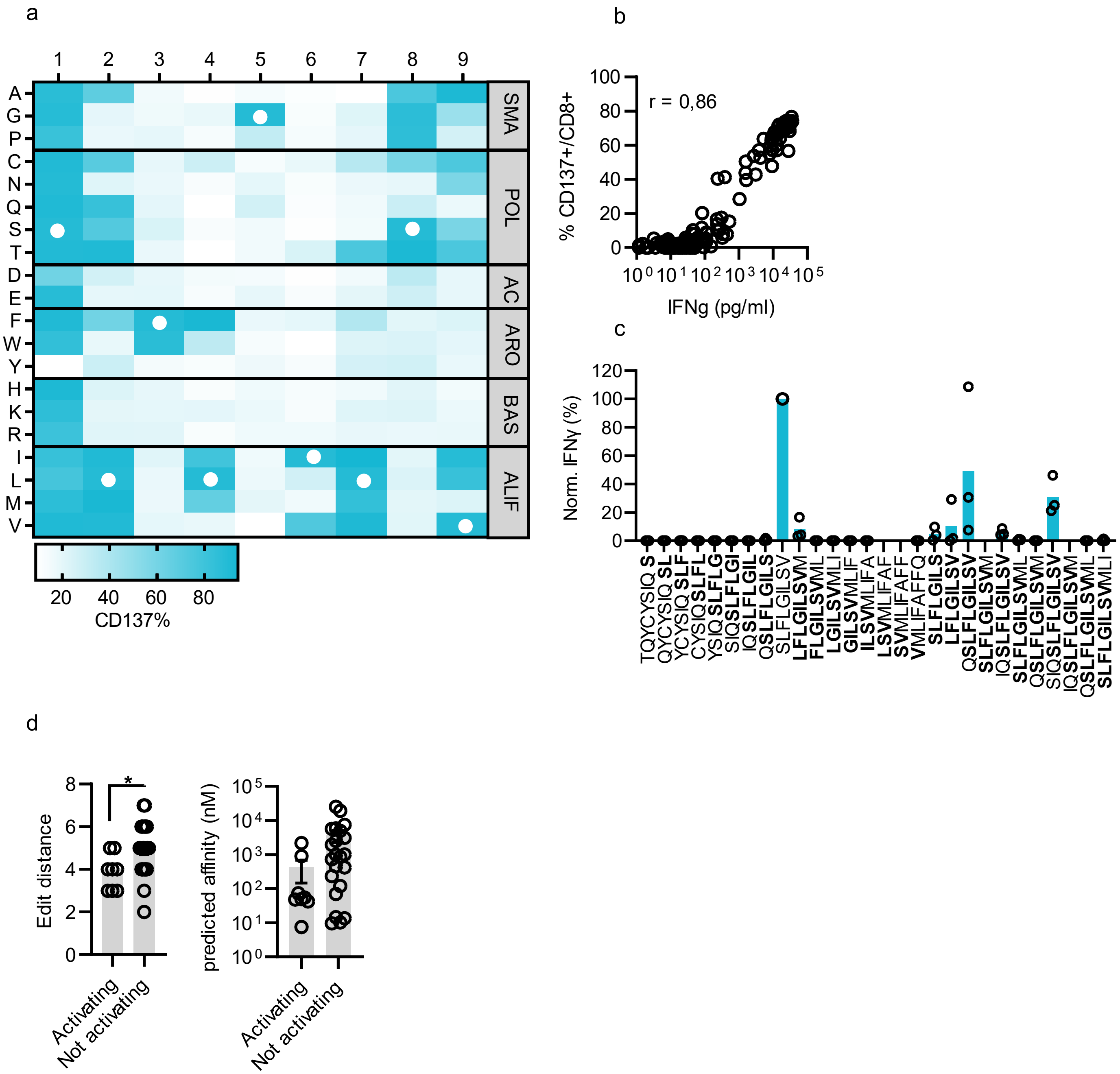

Supplementary Figure 3. A23 TCR-T activation in response to positional scanning peptide matrix and length variants of the cognate peptide

(A) A23 TCR-Ts were incubated with HLA-A2⁺ K562 cells loaded with a library of 9-mer peptides containing single amino acid exchanges compared to the original SLF peptide. Cells from 24 h co-cultures were analysed for CD137 upregulation by flow cytometry. The heat map shows the mean of three independent experiments. Column/row intersections indicate the replaced amino acid at a given position and white circles indicate the original peptide sequence. Substitutions are divided by physicochemical properties: SMA: small; POL: polar; AC: acidic; ARO: aromatic; BAS: basic; ALIF: aliphatic.

(B) Correlation analysis of the percentage of cells expressing CD137 (shown in Supplementary Figure 2A) with IFN-γ release (shown in Figure 1D). r = Pearson correlation coefficient.

(C) A23 TCR-Ts were incubated with HLA-A2⁺ K562 cells loaded with peptides that are upstream or downstream of the original epitope, or longer peptides containing the SLF sequence, at 10^{-7} M concentration. Supernatants of 24 h co-cultures were analysed for IFN-γ content by ELISA. The graphs show pooled data from 3 independent experiments with three technical replicates in each. Dots represent means of technical replicates. Values are normalized to IFN-γ production induced by the original target peptide (range 25,000 – 40,000pg/ml).

(D) Edit distances (left) and predicted binding affinities to HLA-A2 (right), for of A23-activating and non-activating peptides. P-values were determined using Student`s t-test. Dots represent individual peptides with error bars showing mean +/- SD.

Supplementary Figure 4

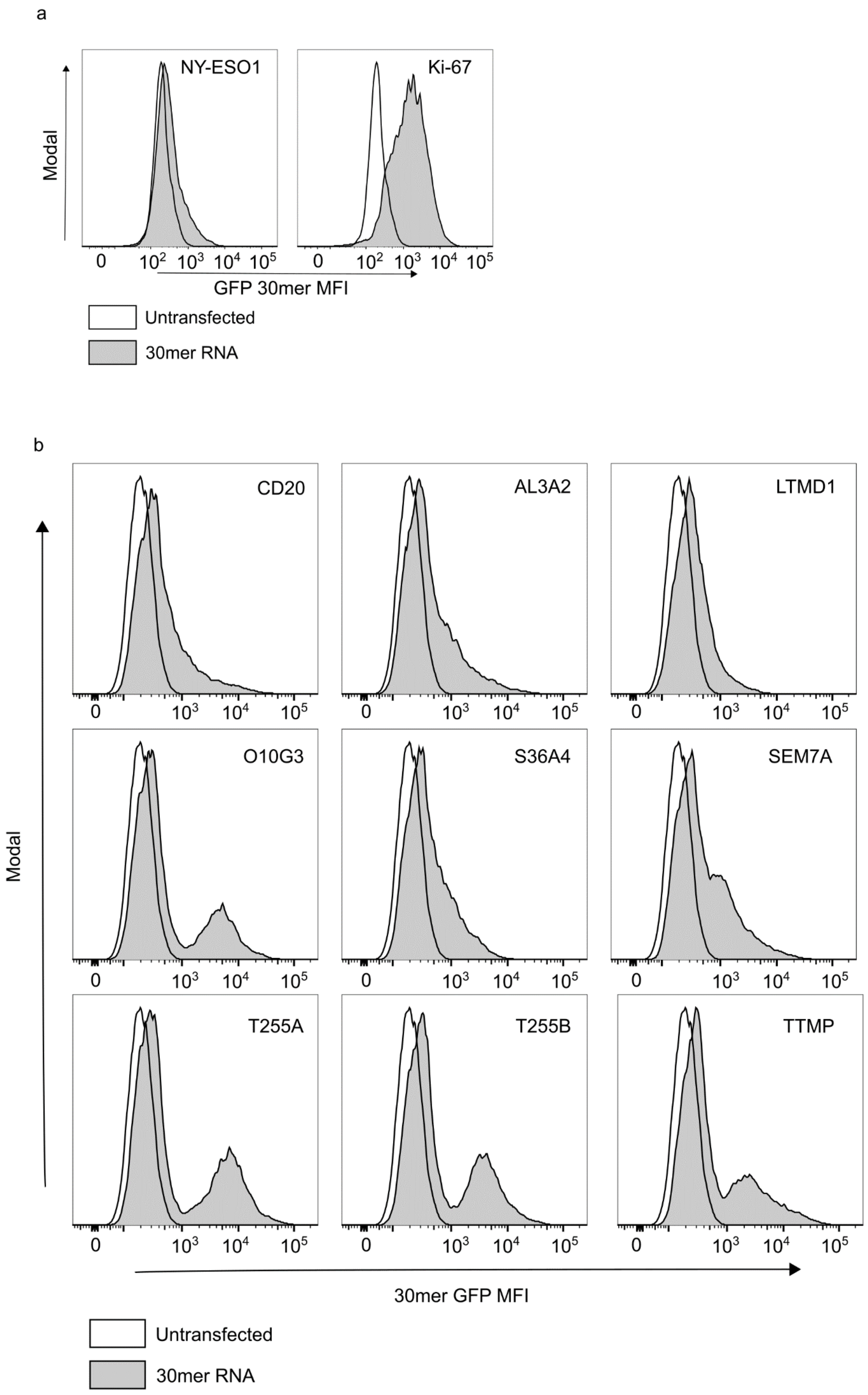

Supplementary Figure 4. GFP expression in cell lines electroporated with mRNA constructs encoding candidate cross-reactive epitopes

mRNA constructs encoding 30 AA long peptides containing a 1G4 **(A)** or A23 **(B)** potentially cross-reactive 9-mer in the middle were electroporated into EBV-LCLs (A) or K562 cells (B). Histograms show MFI of the green fluorescent protein (GFP) tag that was used to control for transfection efficiency.

Supplementary Figure 5

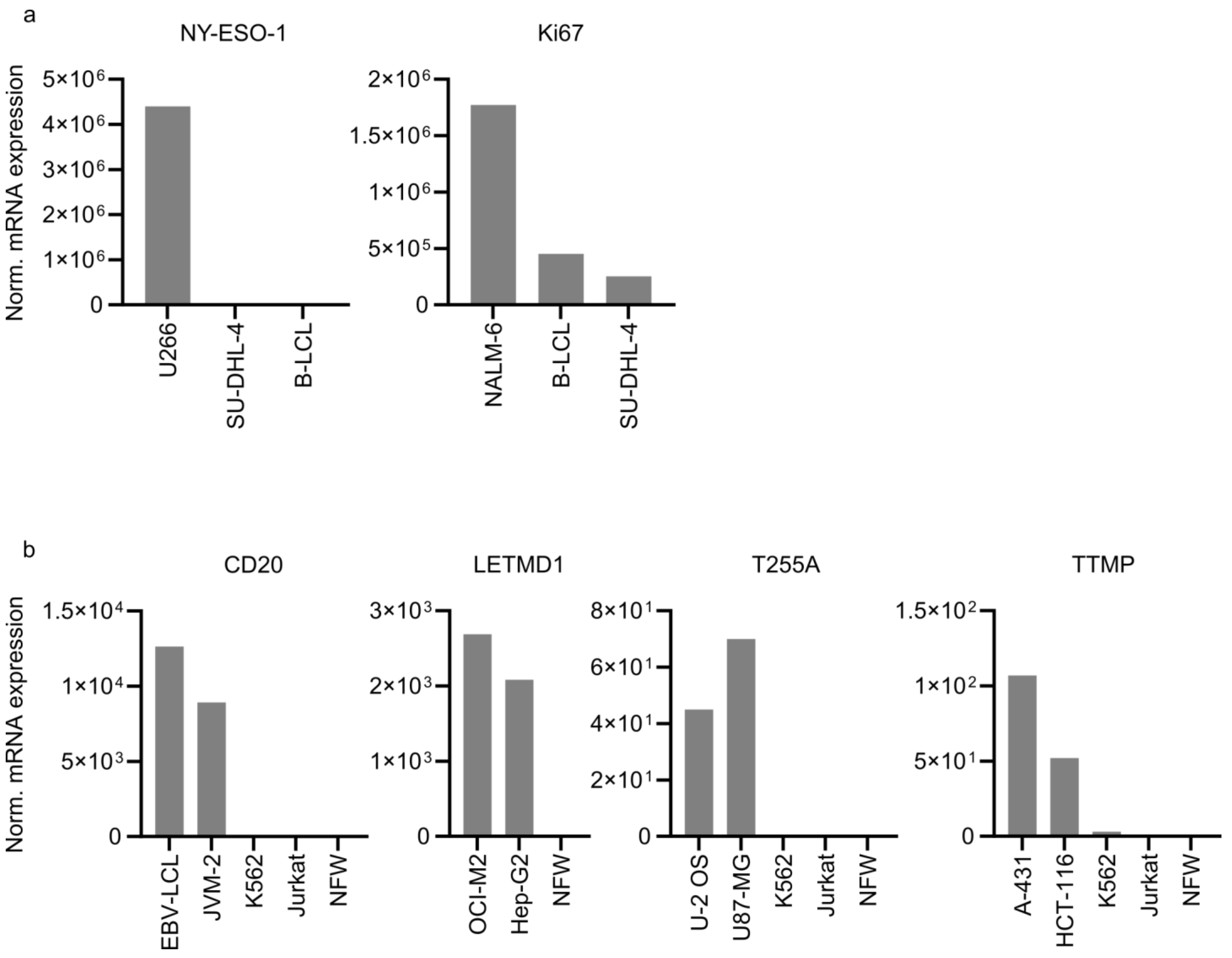

Supplementary Figure 5. RNA expression of candidate cross-reactive proteins in cell lines

Expression of NY-ESO-1 and Ki67 **(A)** and CD20, LETMD1, TTMP and T255A **(B)** mRNA in cell lines as determined by qPCR. The 2^dCt method was employed for normalization and GAPDH or β_2 microglobulin were used as reference genes.

Supplementary Figure 6

a

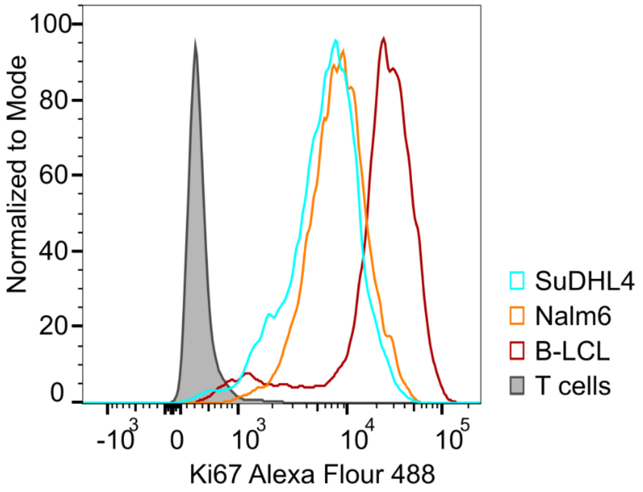

b

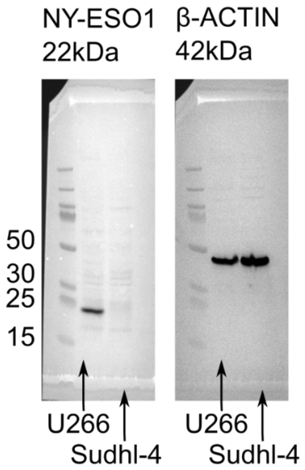

c

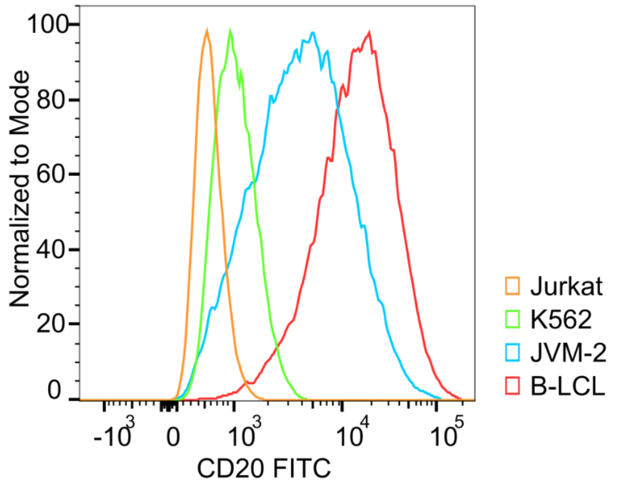

d

Protein (peptide)	A431	HCT116	HepG2	Jurkat	K652	OCIM2	U2OS	U87MG
TTMP (MDLAQPSQPVDELELSVLER)		+						
LETMD1 (VIPLISDAGLR)				+			+	
KI67 (LDLLGNLPGSK)	+	+	+	+	+	+	+	+

Supplementary Figure 6. Protein expression of candidate cross-reactive proteins in cell lines

(A) Expression of Ki67 in cell lines (in culture) and T cells (directly after thawing) as determined by flow cytometry.

(B) Expression of NY-ESO-1 in U266 and Sudhl-4 cell as determined by Western-blot. B-actin used as a house-keeping gene. Blots derive from the same experiment and were processed in parallel.

(C) Expression of CD20 in cell lines as determined by flow cytometry.

(D) Expression of LETMD1, TTMP and Ki67 in cell lines as determined by mass spectrometry using whole-cell lysates and analyzed with the shotgun DDA method.

Supplementary Figure 7

HLA-A*01:01	HLA-A*02:01	HLA-A*03:01	HLA-A*11:01	HLA-A*24:02	HLA-B*40:01	HLA-B*44:02	HLA-B*07:02
		KAFMGTPVQK	TSLPPLPFK	NYGTGMERW	**TEIHNEPFLTL**	**TEIHNEPFLTLW**	TPSAGKAML
		IIFVGTPVQK	IIFVGTPVQK		KEEPLAVSKL	HEQEAILHNF	
		TSLPPLPFK	SVIDEPVRLK		GEEKDINTFL	SEANLIVAKSW	
		VMHTPPVLK	RIQLPVVSK		TEIHNEPFL	SENLLGKQF	
		RIQLPVVSK	LVMHTPPVLK		AEQQITEVFVL	AEQQITEVFVL	
		SVIDEPVRLK			TEVFVLAERI	AEQQITEVF	
					IEIHEQEAIL	AEALEDLVGF	
						KEIERPFETY	

Supplementary Figure 7. Ki67-derived peptides identified by MS of eluted HLA ligands from mono-allelic B721.221 cell lines

Ki67-derived peptides identified during the immunopeptidomics profiling of HLA mono-allelic B721.221 cells by mass-spectrometry. The two peptides overlapping with the FLTLWLTQV-Ki67 sequence are displayed in bold.

Supplementary Figure 8

Alignment of HLA-A*02:01, HLA-A*02:03, HLA-A*02:06, and HLA-A*02:7

```
A*02_01_01_01    MAVMAPRTLVLLLSGALALTQTWAGSHSMRYFFTSVSRPGRGEPRFIAVGYVDDTQFVRF   60
A*02_03_01_01    MAVMAPRTLVLLLSGALALTQTWAGSHSMRYFFTSVSRPGRGEPRFIAVGYVDDTQFVRF   60
A*02_06_01_01    MAVMAPRTLVLLLSGALALTQTWAGSHSMRYFYTSVSRPGRGEPRFIAVGYVDDTQFVRF   60
A*02_07_01_01    MAVMAPRTLVLLLSGALALTQTWAGSHSMRYFFTSVSRPGRGEPRFIAVGYVDDTQFVRF   60
                 ********************************:***************************

A*02_01_01_01    DSDAASQRMEPRAPWIEQEGPEYWDGETRKVKAHSQTHRVDLGTLRGYYNQSEAGSHTVQ   120
A*02_03_01_01    DSDAASQRMEPRAPWIEQEGPEYWDGETRKVKAHSQTHRVDLGTLRGYYNQSEAGSHTVQ   120
A*02_06_01_01    DSDAASQRMEPRAPWIEQEGPEYWDGETRKVKAHSQTHRVDLGTLRGYYNQSEAGSHTVQ   120
A*02_07_01_01    DSDAASQRMEPRAPWIEQEGPEYWDGETRKVKAHSQTHRVDLGTLRGYYNQSEAGSHTVQ   120
                 ******:***************************************************:*

A*02_01_01_01    RMYGCDVGSDWRFLRGYHQYAYDGKDYIALKEDLRSWTAADMAAQTTKHKWEAAHVAEQL   180
A*02_03_01_01    RMYGCDVGSDWRFLRGYHQYAYDGKDYIALKEDLRSWTAADMAAQTTKHKWETAHEAEQW   180
A*02_06_01_01    RMYGCDVGSDWRFLRGYHQYAYDGKDYIALKEDLRSWTAADMAAQTTKHKWEAAHVAEQL   180
A*02_07_01_01    RMCGCDVGSDWRFLRGYHQYAYDGKDYIALKEDLRSWTAADMAAQTTKHKWEAAHVAEQL   180
                 **:*************************************************:**.***.

A*02_01_01_01    RAYLEGTCVEWLRRYLENGKETLQRTDAPKTHMTHHAVSDHEATLRCWALSFYPAEITLT   240
A*02_03_01_01    RAYLEGTCVEWLRRYLENGKETLQRTDAPKTHMTHHAVSDHEATLRCWALSFYPAEITLT   240
A*02_06_01_01    RAYLEGTCVEWLRRYLENGKETLQRTDAPKTHMTHHAVSDHEATLRCWALSFYPAEITLT   240
A*02_07_01_01    RAYLEGTCVEWLRRYLENGKETLQRTDAPKTHMTHHAVSDHEATLRCWALSFYPAEITLT   240
                 ************************************************************

A*02_01_01_01    WQRDGEDQTQDTELVETRPAGDGTFQKWAAVVVPSGQEQRYTCHVQHEGLPKPLTLRWEP   300
A*02_03_01_01    WQRDGEDQTQDTELVETRPAGDGTFQKWAAVVVPSGQEQRYTCHVQHEGLPKPLTLRWEP   300
A*02_06_01_01    WQRDGEDQTQDTELVETRPAGDGTFQKWAAVVVPSGQEQRYTCHVQHEGLPKPLTLRWEP   300
A*02_07_01_01    WQRDGEDQTQDTELVETRPAGDGTFQKWAAVVVPSGQEQRYTCHVQHEGLPKPLTLRWEP   300
                 ************************************************************

A*02_01_01_01    SSQPTIPIVGIIAGLVLFGAVITGAVVAAVMWRRKSSDRKGGSYSQAASSDSAQGSDVSL   360
A*02_03_01_01    SSQPTIPIVGIIAGLVLFGAVITGAVVAAVMWRRKSSDRKGGSYSQAASSDSAQGSDVSL   360
A*02_06_01_01    SSQPTIPIVGIIAGLVLFGAVITGAVVAAVMWRRKSSDRKGGSYSQAASSDSAQGSDVSL   360
A*02_07_01_01    SSQPTIPIVGIIAGLVLFGAVITGAVVAAVMWRRKSSDRKGGSYSQAASSDSAQGSDVSL   360
                 ************************************************************

A*02_01_01_01    TACKV   365
A*02_03_01_01    TACKV   365
A*02_06_01_01    TACKV   365
A*02_07_01_01    TACKV   365
                 *****
```

Supplementary Figure 8. Alignment of the amino acid sequences of HLA-A*02:01, -A*02:03, -A*02:06, and -A*02:07

Sequence alignment showing that conserved contacts formed between HLA-A*02 residues and αβ TCRs are identical in depicted HLA-A*02 alleles (red). Differences to HLA-A*02:01 are shown in blue.

Supplementary Figure 9

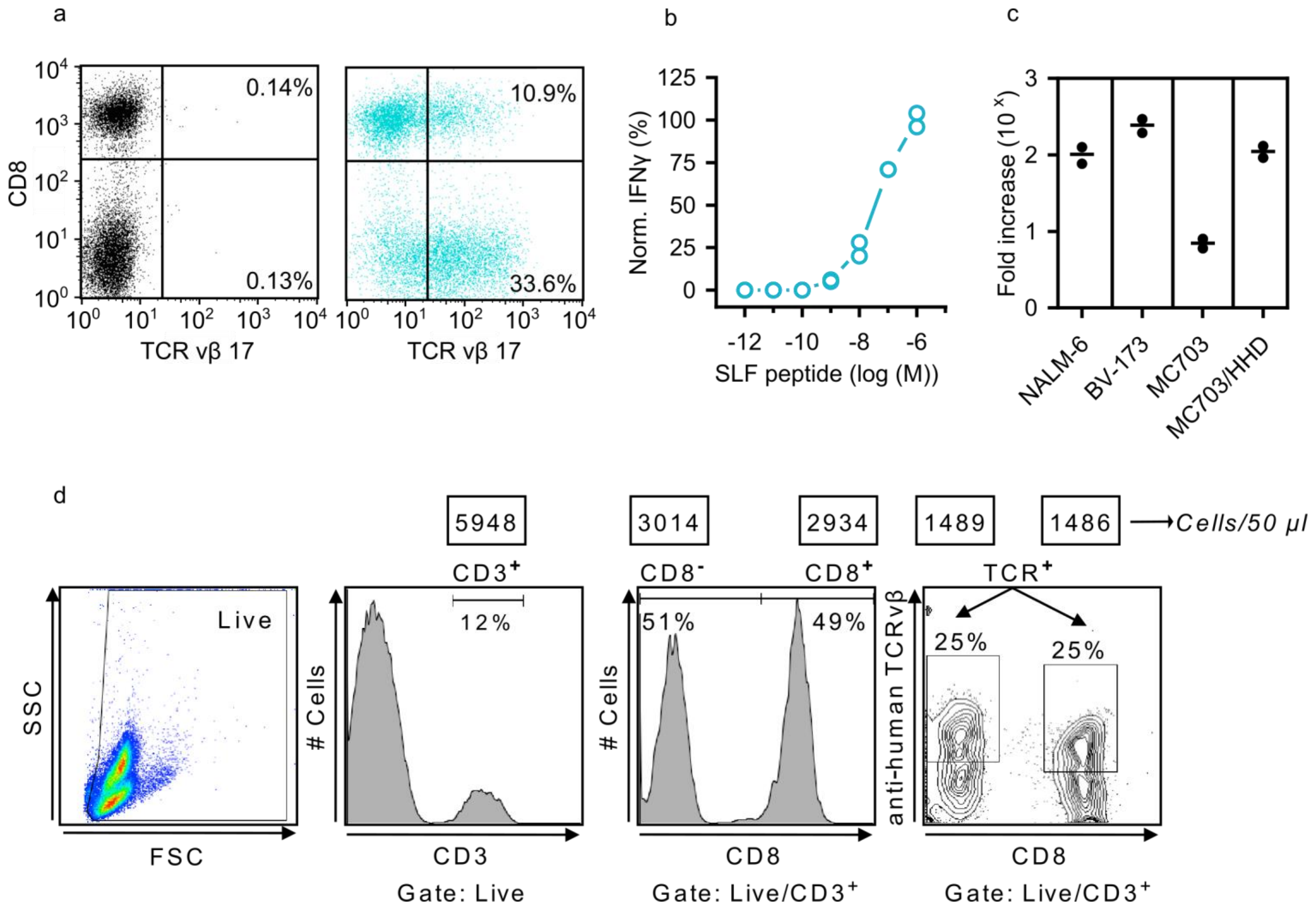

Supplementary Figure 9. A23 TCR is expressed on splenocytes of HHD mice and is reactive to the cognate peptide.

(A) T cells were isolated from spleens of HHD mice and retrovirally transduced with TCR-encoding vectors. TCR-expression was determined by flow cytometry 10 days after transduction, using TCRvβ- and CD8-specific antibodies. The percentage of TCR^+CD8^+ T cells is depicted in the upper right quadrant.

(B) TCR-engineered HHD T cells were incubated with irradiated HHD mouse splenocytes loaded with indicated amounts of SLF peptide. Supernatants of 24 h co-cultures were analyzed for IFN-γ content by ELISA. The relative amount of IFN-γ refers to 10^{-6} M of SLF peptide. Data shown are technical replicates and representative of 2 independent experiments.

(C) Expression of HLA-A2 measured by flow cytometry of naturally HLA-A2$^+$ human NALM-6, BV-173 cell lines and MC703 cells generated in HHD mice. To reach HLA-A2 levels comparable to the human cell lines, retroviral transduction of additional HHD transgene was necessary (MC703/HHD).

(D) Gating strategy for assessment of the number of transduced effector cells in a sample. Effector cells were identified as live single cells staining positively with CD3, CD8 and anti-human TCRvβ. Total cells in each sample were measured to determine total cell counts per 50 µl blood.

Supplementary Table 1

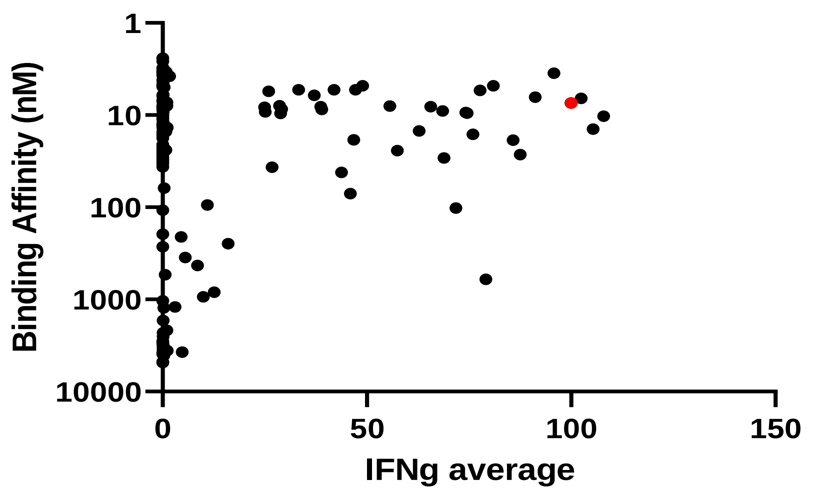

Sequence	Normalized IFNg average	Binding affinity score
ALFLGILSV	65,66217843	8.1
CLFLGILSV	87,55784193	26.8
DLFLGILSV	5,575920782	351.8
ELFLGILSV	71,82433757	102.3
FLFLGILSV	95,78223227	3.5
GLFLGILSV	68,56298101	9.0
HLFLGILSV	105,3632177	14.2
ILFLGILSV	28,61487715	7.9
KLFLGILSV	33,25350107	5.3
LLFLGILSV	24,98537512	8.2
MLFLGILSV	48,9759336	4.8
NLFLGILSV	62,75775618	14.8
PLFLGILSV	16,01384019	249.1
QLFLGILSV	75,98739813	16.1
RLFLGILSV	29,13425642	8.6
TLFLGILSV	74,54996636	9.5
VLFLGILSV	74,23805537	9.4
WLFLGILSV	38,74309684	8.1
SAFLGILSV	4,575310221	209.9
SCFLGILSV	12,65142624	834.6
SDFLGILSV	1,11152542	3602.5
SEFLGILSV	0,139129678	3204.7
SFFLGILSV	9,936097056	936.7
SGFLGILSV	0,104847858	2293.3
SHFLGILSV	0,288353833	4043.7
SIFLGILSV	57,48201431	24.2
SKFLGILSV	0,021307888	2951.8
SMFLGILSV	77,70161842	5.4
SNFLGILSV	0,098758457	3126.9
SPFLGILSV	0,11232253	4026.9
SQFLGILSV	26,81057347	36.8
SRFLGILSV	0,01286841	3815.0
SSFLGILSV	8,580676693	427.8
STFLGILSV	45,94070153	70.8
SVFLGILSV	43,75741924	41.8

SWFLGILSV	0,298527428	1231.3
SYFLGILSV	1,055545942	2168.7
SLALGILSV	0,061990423	10.0
SLCLGILSV	0	36.5
SLDLGILSV	0	12.8
SLELGILSV	0	107.4
SLGLGILSV	0,021961348	31.8
SLHLGILSV	0	30.0
SLILGILSV	0,01701951	10.2
SLKLGILSV	0,003281301	196.1
SLLLGILSV	0,033182512	8.7
SLMLGILSV	0,165420539	4.4
SLNLGILSV	0,025242649	20.9
SLPLGILSV	0,021961348	26.8
SLQLGILSV	0,188434548	30.3
SLRLGILSV	0,015306394	268.3
SLSLGILSV	0,248229782	14.9
SLTLGILSV	0	24.9
SLVLGILSV	0,077263336	23.7
SLWLGILSV	91,23234624	6.4
SLYLGILSV	0,094062277	8.2
SLFAGILSV	0	3.3
SLFCGILSV	0,834226983	3.4
SLFDGILSV	0,060155845	2.4
SLFEGILSV	0	2.6
SLFFGILSV	80,9682604	4.8
SLFGGILSV	0,459197014	3.4
SLFHGILSV	0	3.5
SLFIGILSV	38,93452726	8.7
SLFKGILSV	0,003281301	4.2
SLFMGILSV	25,93599603	5.5
SLFNGILSV	0	3.3
SLFPGILSV	0,073306777	3.1
SLFQGILSV	0	4.8
SLFRGILSV	0,00871731	4.7
SLFSGILSV	0,030212431	3.1
SLFTGILSV	0	3.5
SLFVGILSV	0,190817039	6.9
SLFWGILSV	1,538830737	3.8
SLFYGILSV	0	3.7
SLFLAILSV	0	12.5
SLFLCILSV	0,048581164	12.2
SLFLDILSV	0,029930223	8.6
SLFLEILSV	0,016875264	11.1
SLFLFILSV	0	10.1
SLFLHILSV	0	8.3
SLFLIILSV	0,073569549	13.4
SLFLKILSV	0,120088257	12.9
SLFLLILSV	0,04234132	13.2
SLFLMILSV	0,050157034	10.3
SLFLNILSV	0,161918455	12.0
SLFLPILSV	0,75886321	15.1
SLFLQILSV	0,184499787	11.8
SLFLRILSV	0,043992942	17.7
SLFLSILSV	0,149352612	11.1
SLFLTILSV	0,020000313	16.4
SLFLWILSV	0	6.1

SLFLYILSV	0,068961136	6.2
SLFLGALSV	0,00456621	15.4
SLFLGCLSV	0,207139131	13.4
SLFLGDLSV	0	11.1
SLFLGELSV	0,003281301	12.1
SLFLGFLSV	0,055236118	10.7
SLFLGGLSV	0	27.1
SLFLGHLSV	0	15.4
SLFLGKLSV	0,048581164	22.3
SLFLGLLSV	0,38215147	7.9
SLFLGMLSV	0,045415813	8.3
SLFLGNLSV	0	8.5
SLFLGPLSV	0,101820795	9.2
SLFLGQLSV	0,056324879	9.7
SLFLGRLSV	0	33.6
SLFLGSLSV	0	9.3
SLFLGTLSV	1,064428276	7.3
SLFLGVLSV	25,10681133	9.2
SLFLGYLSV	0	11.3
SLFLGIASV	0,00865144	7.0
SLFLGICSV	1,053227292	7.9
SLFLGIDSV	0	10.2
SLFLGIESV	0,041719402	9.8
SLFLGIFSV	0,290905734	3.7
SLFLGIGSV	0,083096538	22.1
SLFLGIHSV	0,046406975	4.9
SLFLGIISV	102,4260427	6.6
SLFLGIKSV	0,022910898	26.4
SLFLGIMSV	41,9867016	5.3
SLFLGINSV	0,018281536	8.2
SLFLGIPSV	0,037031829	3.4
SLFLGIQSV	0,055782122	8.0
SLFLGIRSV	0,113094878	25.0
SLFLGISSV	0,089958072	7.4
SLFLGITSV	37,11996828	6.1
SLFLGIVSV	99,94318288	7.4
SLFLGIWSV	0,12227345	3.1
SLFLGIYSV	1,715298723	3.8
SLFLGILAV	28,84076829	9.6
SLFLGILCV	1,111529107	13.6
SLFLGILDV	0,751552831	23.9
SLFLGILEV	0,12609572	6.0
SLFLGILFV	0,079219988	7.0
SLFLGILGV	55,62532235	8.0
SLFLGILHV	0,403211733	10.9
SLFLGILIV	0,027644676	28.8
SLFLGILKV	0,022969109	13.0
SLFLGILLV	0,22724709	11.9
SLFLGILMV	0,120306069	14.5
SLFLGILNV	0,027656682	9.5
SLFLGILPV	47,18036887	5.3
SLFLGILQV	0,242935786	13.3
SLFLGILRV	0	13.5
SLFLGILTV	107,9515958	10.3
SLFLGILVV	0	20.9
SLFLGILWV	0,092933442	9.4
SLFLGILYV	0,414148219	5.0

SLFLGILSA	68,84737693	29.1
SLFLGILSC	79,15731149	607.2
SLFLGILSD	0,004218816	4778.2
SLFLGILSE	0,043628572	3903.4
SLFLGILSF	0,008906389	1033.1
SLFLGILSG	3,016711123	1209.2
SLFLGILSH	0,046406975	4854.0
SLFLGILSI	85,76850666	18.7
SLFLGILSK	0,018281536	3743.4
SLFLGILSL	46,76174769	18.5
SLFLGILSM	0,338363576	61.9
SLFLGILSN	4,773542615	3737.5
SLFLGILSP	0,125151205	1687.0
SLFLGILSQ	0,018281536	2851.8
SLFLGILSR	0,128928404	3497.6
SLFLGILSS	0,660947827	539.0
SLFLGILST	10,93775825	94.4
SLFLGILSW	0,083907561	2525.2
SLFLGILSY	0,134659844	3358.7
SLFLGILSV	100	7.4

Supplementary Table 1 Predicted binding affinities of all A23 TCR peptide mimotopes used in our study.

Correlation of IFN-γ production and predicted binding affinity is shown, with the cognate target peptide marked in red.

Supplementary Table 2

Gene	Sequence	Edit distance	nM	Rank	Binding strength	Cut-off
KI67	**F**L**TL**W**L**TQ**V**	5	6.1	0.050	SB	10 %
FGRL1	**T**LL**L**W**LC**Q**A**	5	121.7	1.200	WB	
NY-ESO1	SLLMWITQC	0	666.7	3.500		
TM245	**V**L**DL**W**L**TQ**G**	5	5508.9	10.000		
AT135	**T**L**TL**W**LS**Q**G**	6	11434.4	17.000		
AGRB3	SL**GT**W**S**TQ**G**	4	15177.0	21.000		
CSN2	**A**L**DK**W**TN**Q**L**	6	80.3	0.900	WB	5 %
TMUB1	**L**LL**L**W**YC**Q**I**	5	85.3	1.000	WB	
PPR37	**T**L**VL**W**NN**Q**L**	6	403.1	2.500		
F231L	**I**LL**V**W**NS**Q**T**	5	796.3	3.500		
DXO	**K**LL**K**W**WA**Q**S**	5	1214.6	4.500		
PLSI	**IIIK**W**VN**Q**T**	7	3204.9	7.500		

Supplementary Table 2 List of candidate off-target peptides for 1G4 TCR based on TCR-fingerprint

List of potentially 1G4 TCR cross-reactive peptides identified in a search using ScanProSite tool with data generated from the peptide library screen presented in Fig. 1A. Edit distance is the minimal number of amino acid exchanges that is needed to reach the original target sequence. HLA-A2 binding affinity (nM) and Rank was predicted by NetMHC 4.0. SB – predicted strong binder, WB- predicted weak binder.

Supplementary Table 3

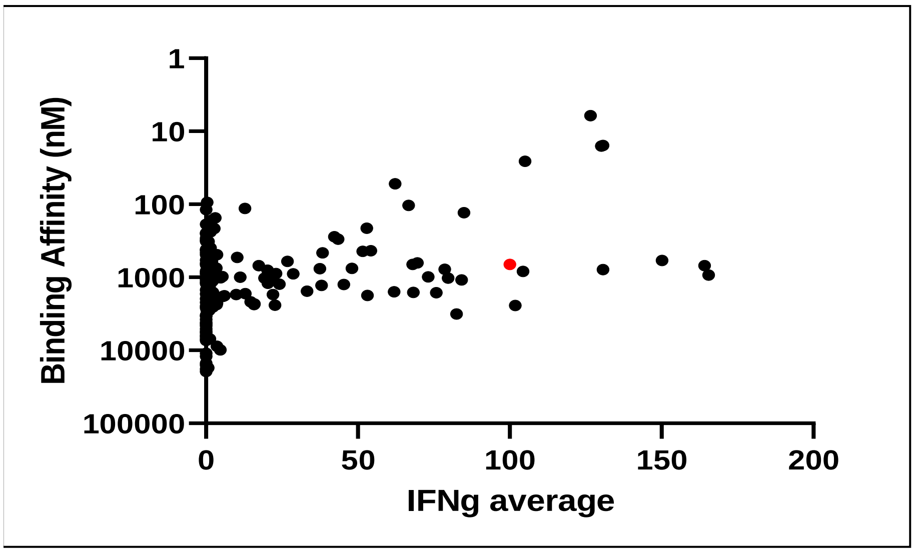

**Sequence**	Normalized IFNg average	Binding affinity score
ALLMWITQC	**48,10**	755.5
CLLMWITQC	**33,26**	1550.1
DLLMWITQC	**0,00**	6540.5
ELLMWITQC	**0,00**	4639.0
FLLMWITQC	**12,82**	113.6
GLLMWITQC	**73,14**	987.3
HLLMWITQC	**45,41**	1250.1
ILLMWITQC	**26,82**	606.5
KLLMWITQC	**54,28**	431.5
LLLMWITQC	**68,01**	668.2
MLLMWITQC	**42,24**	277.9
NLLMWITQC	**0,00**	1494.6
PLLMWITQC	**0,00**	6996.8
QLLMWITQC	**75,87**	1617.1
RLLMWITQC	**78,56**	777.9
TLLMWITQC	**84,21**	1080.6
VLLMWITQC	**104,40**	828.1
WLLMWITQC	**20,27**	801.3
YLLMWITQC	**84,96**	129.8
SALMWITQC	**3,65**	8746.3
SCLMWITQC	**4,72**	9890.7
SDLMWITQC	**0,00**	19388.6
SELMWITQC	**0,70**	17367.0
SFLMWITQC	**0,00**	10994.1
SGLMWITQC	**0,00**	15853.9
SHLMWITQC	**0,00**	19281.3
SILMWITQC	**82,49**	3181.5
SKLMWITQC	**0,00**	18020.7
SMLMWITQC	**150,20**	589.0
SNLMWITQC	**0,00**	19019.8
SPLMWITQC	**0,00**	18632.0
SQLMWITQC	**0,00**	4375.8
SRLMWITQC	**0,00**	18782.0

SSLMWITQC	**0,00**	10551.3
STLMWITQC	**0,00**	6214.3
SVLMWITQC	**0,00**	4565.1
SWLMWITQC	**0,00**	12097.5
SYLMWITQC	**0,00**	15096.6
SLAMWITQC	**79,73**	1027.2
SLCMWITQC	**53,17**	1763.7
SLDMWITQC	**38,02**	1295.0
SLEMWITQC	**0,00**	4565.4
SLFMWITQC	**51,65**	438.9
SLGMWITQC	**22,67**	2406.9
SLHMWITQC	**15,86**	2366.1
SLIMWITQC	**130,77**	786.1
SLKMWITQC	**0,16**	6321.2
SLMMWITQC	**52,91**	211.7
SLNMWITQC	**14,75**	2170.3
SLPMWITQC	**0,00**	2486.5
SLQMWITQC	**101,83**	2439.3
SLRMWITQC	**0,00**	5773.3
SLSMWITQC	**61,90**	1583.7
SLTMWITQC	**15,91**	2304.8
SLVMWITQC	**68,23**	1603.1
SLWMWITQC	**43,48**	300.0
SLYMWITQC	**0,00**	582.8
SLLAWITQC	**0,00**	309.7
SLLCWITQC	**0,83**	325.3
SLLDWITQC	**0,34**	93.7
SLLEWITQC	**0,00**	117.9
SLLFWITQC	**0,00**	639.9
SLLGWITQC	**0,00**	322.1
SLLHWITQC	**0,00**	414.0
SLLIWITQC	**0,00**	1033.4
SLLKWITQC	**164,17**	687.9
SLLLWITQC	**165,51**	933.4
SLLNWITQC	**0,00**	316.1
SLLPWITQC	**0,00**	291.4
SLLQWITQC	**69,56**	636.5
SLLRWITQC	**0,00**	906.1
SLLSWITQC	**0,00**	292.1
SLLTWITQC	**38,38**	464.1
SLLVWITQC	**19,30**	1028.2
SLLWWITQC	**0,00**	424.4
SLLYWITQC	**0,00**	491.2
SLLMAITQC	**0,68**	1973.7
SLLMCITQC	**1,66**	1015.1
SLLMDITQC	**1,84**	1896.6
SLLMEITQC	**1,04**	2313.0
SLLMFITQC	**0,00**	820.0
SLLMGITQC	**0,00**	1683.4
SLLMHITQC	**1,12**	1787.5
SLLMIITQC	**0,00**	1219.5
SLLMKITQC	**0,00**	2615.7
SLLMLITQC	**0,00**	1159.3
SLLMMITQC	**1,21**	1334.9
SLLMNITQC	**0,00**	2440.2

SLLMPITQC	**0,27**	2822.5
SLLMQITQC	**1,23**	2571.5
SLLMRITQC	**2,24**	2556.1
SLLMSITQC	**0,00**	1961.6
SLLMTITQC	**0,00**	1699.3
SLLMVITQC	**0,00**	1211.1
SLLMYITQC	**1,05**	731.6
SLLMWATQC	**0,00**	2217.2
SLLMWCTQC	**0,00**	1175.1
SLLMWDTQC	**1,36**	2286.9
SLLMWETQC	**0,00**	2801.6
SLLMWFTQC	**2,54**	1077.1
SLLMWGTQC	**0,00**	3763.9
SLLMWHTQC	**0,00**	3407.0
SLLMWKTQC	**0,00**	5482.2
SLLMWLTQC	**37,45**	758.9
SLLMWMTQC	**21,68**	1045.8
SLLMWNTQC	**5,59**	1818.3
SLLMWPTQC	**2,29**	1605.2
SLLMWQTQC	**0,87**	2202.7
SLLMWRTQC	**0,00**	5968.1
SLLMWSTQC	**22,01**	1715.0
SLLMWTTQC	**24,14**	1246.7
SLLMWVTQC	**28,77**	893.8
SLLMWWTQC	**9,91**	1721.5
SLLMWYTQC	**6,00**	1792.1
SLLMWIAQC	**10,29**	534.3
SLLMWICQC	**23,08**	886.1
SLLMWIDQC	**20,44**	1205.8
SLLMWIEQC	**2,90**	1010.7
SLLMWIFQC	**2,68**	214.4
SLLMWIGQC	**3,62**	2258.9
SLLMWIHQC	**0,00**	484.2
SLLMWIIQC	**0,00**	570.1
SLLMWIKQC	**1,04**	2831.5
SLLMWILQC	**0,00**	673.5
SLLMWIMQC	**0,00**	458.6
SLLMWINQC	**5,37**	976.4
SLLMWIPQC	**0,00**	253.4
SLLMWIQQC	**4,79**	1015.5
SLLMWIRQC	**0,00**	2622.9
SLLMWISQC	**11,28**	993.5
SLLMWIVQC	**3,40**	748.8
SLLMWIWQC	**1,76**	166.2
SLLMWIYQC	**0,00**	248.7
SLLMWITAC	**0,00**	419.9
SLLMWITCC	**1,74**	558.9
SLLMWITDC	**0,00**	1081.6
SLLMWITEC	**1,54**	237.9
SLLMWITFC	**0,00**	251.5
SLLMWITGC	**1,53**	397.6
SLLMWITHC	**3,62**	489.3
SLLMWITIC	**0,00**	1066.4
SLLMWITKC	**0,00**	831.4
SLLMWITLC	**0,00**	472.1

SLLMWITMC	**1,96**	607.0
SLLMWITNC	**0,00**	457.8
SLLMWITPC	**0,00**	187.9
SLLMWITRC	**0,00**	675.6
SLLMWITSC	**0,00**	311.4
SLLMWITTC	**1,46**	399.2
SLLMWITVC	**0,00**	864.0
SLLMWITWC	**0,00**	437.4
SLLMWITYC	**3,11**	153.4
SLLMWITQA	**105,04**	25.7
SLLMWITQD	**0,00**	7331.1
SLLMWITQE	**0,00**	5932.6
SLLMWITQF	**2,04**	1138.1
SLLMWITQG	**12,89**	1666.0
SLLMWITQH	**1,29**	6963.6
SLLMWITQI	**130,18**	16.0
SLLMWITQK	**0,00**	5266.9
SLLMWITQL	**130,75**	15.6
SLLMWITQM	**62,28**	52.1
SLLMWITQN	**0,64**	5502.5
SLLMWITQP	**3,46**	2361.3
SLLMWITQQ	**0,00**	4169.6
SLLMWITQR	**0,00**	5076.7
SLLMWITQS	**17,35**	690.1
SLLMWITQT	**66,77**	103.5
SLLMWITQV	**126,64**	6.1
SLLMWITQW	**0,00**	3312.0
SLLMWITQY	**0,00**	4299.4
SLLMWITQC	**100,00**	666.7

Supplementary Table 3 Predicted binding affinities of all 1G4 TCR peptide mimotopes used in the study.

Correlation of IFN-γ production and predicted binding affinity is shown, with the cognate target peptide marked in red.

Supplementary Table 4

Gene	Sequence	Edit distance	nM	Rank	Binding strength	Cut-off
CD20	SLFLGILSV	0	7.4	0.070	SB	10 %
CD6	WLFFGITGL	5	9.6	0.125	SB	
S52A3	LLFLGVLSV	2	10.3	0.125	SB	
ARAID	LMFFGILGA	5	13.5	0.175	SB	
BTNL8	GLFFGIVGL	5	14.7	0.200	SB	
TTMP	SIFLGVITV	4	42.6	0.600	WB	
S36A4	LVFIGIISV	4	48.3	0.600	WB	
T255A/ T255B	GLFLGIITA	4	50.1	0.600	WB	
SEM7A	SLWLGVLPT	3	54.9	0.700	WB	
LETMD1	CLFLGIISI	3	73.4	0.900	WB	
MSH3	NIFIGIVGV	5	119.3	1.200	WB	
AP5S1	VVWLGVLSL	5	232.8	1.800	WB	
ABCA2	NLFIGITAT	5	459.0	3.000		
FETUA	HTFMGVVSL	6	735.3	3.500		
AL3A2	LTFLGIVAA	5	902.7	4.000		
S35F6	SQWLGILAT	4	1005.0	4.000		
PAXI1	QIFFGITAC	6	5820.1	11.000		
MRP2	TCFLGIIST	4	7437.8	13.000		
CML1	VCFLGILGN	4	19066.0	26.000		
O10G3	YIFLGVLSV	3	7.6	0.070	SB	5 %
MARH6	FAWLGVVPL	7	70.3	0.800	WB	
CNR1	MFWIGVTSV	6	422.9	2.500		
CYAC3	HSWLGITTV	5	865.6	4.000		
POK18	VSFLGVTTV	5	991.2	4.000		
CHAD	GAFLGVTTL	6	1949.0	6.000		
T255B/2	GSFLGIIGI	5	2163.9	6.000		
O10R2	YFFLGILST	3	2497.5	6.500		
P4HA2	MAWFGVLSC	6	3115.4	7.500		
SEM4F	GFFLGILAA	4	4939.8	9.500		
GPR85	IAFLGVLSC	4	5689.9	11.000		
S15A3	AAFFGVTAN	7	25125.3	36.000		

Supplementary Table 4 List of candidate off-target peptides for A23 TCR based on TCR-fingerprint

List of potentially A23 TCR cross-reactive peptides identified in a search using ScanProSite tool with data generated from the peptide library presented in Fig. 1D. Edit distance is the minimal number of amino acid exchanges that is needed to reach the original target sequence. HLA-A2 binding affinity (nM) and Rank was predicted by NetMHC 4.0. SB – predicted strong binder, WB- predicted weak binder.

Supplementary Table 5

	IHW ID	Alternative ID	HLA type						Origin
			A		B		C		
1	1136	1362-8575	0101	1101	0702	5101	0702	1502	FHCRC
2	1170	1413-1218	6801	1101	1302	0702	0303	0702	FHCRC
3	1185	1416-1337	0205	2501	4901	4402	0701	0501	FHCRC
4	9383	FH9	2402	3303	4801	4403	0801	0701	FHCRC
5	9387	FH13	3402	6802	4403	1510	0401	030402	FHCRC
6	9401	TER-259	3201	6802	0801	4402	0102	07	FHCRC
7	9423	FH36	3402	7401	0801	1503	0701	0202	FHCRC
8	9431	FH43	3001	3301	5301	8101	04	08	FHCRC
9	9441	FH53	2403	2407	5106	3505	0401	1204	FHCRC
10	9453	FH65	29	30	5301	4501	04	0602	FHCRC
11	9465	SCL-116A	0301	3002	1801	5601	0102	05	FHCRC
12	9466	FH76	2402	2402	5501	5501	0102	0102	FHCRC
13	9010	AMAI	6802	6802	5301	5301	0401	0401	FHCRC
14	9040	BM15	0101	0101	4901	4901	0701	0701	FHCRC
15	9043	BM21	0101	0101	4101	4101	1701	1701	FHCRC
16	9044	BRIP	2402	2402	5101	1517	0701	1504	FHCRC
17	9234	CRB	6602	3002	1801	5801	0701	0701	ECACC
18	9366	DAUDI	0102	6601	5801	5802	0602	0302	ECACC
19	9210	DK1	0203	3301	4403	4001	0304	14	ECACC
20	9080	EHM	0301	0301	3501	3503	0401	0401	FHCRC
21	9105	FPAF	0101	0101	3502	3502	0401	0401	FHCRC
22	9363	GRC-187	6801	3101	3511	1504	0303	0304	ECACC
23	9005	HOM-2	0301	0301	2705	2705	0102	0102	FHCRC
24	9009	KASOII	0101	0101	3701	3701	0602	0602	FHCRC
25	9073	KT12	2402	3101	3501	5101	0401	1202	FHCRC
26	9107	KT3	2402	2402	5401	5401	0102	0102	FHCRC
27	9226	MOLT-4	0101	2501	5701	1801	0602	1203	ECACC
28	9100	OLGA	3101	3101	1501	1520	0102	0304	FHCRC
29	9028	PE117	2402	2402	4001	4002	0304	0304	FHCRC
30	9021	RSH	6802	3001	4201	4201	1701	1701	FHCRC
31	9076	T7526	0206	0207	4601	4601	0102	0801	FHCRC
32	9042	TISI	2402	2402	3508	3508	0401	0401	FHCRC
33	9029	WT51	2301	2301	1401	1401	0802	0802	FHCRC

Supplementary Table 5 B-LCL cell lines with known HLA types included in the panel used for assessment of TCR-reactivity to unintended HLA alleles

Supplementary Table 6

USA NMDP European Caucasian (n=1 242 890)					
A		B		C	
Allele	Freqency	Allele	Freqency	Allele	Freqency
A*02:01	0,276	B*07:02	0,131	C*07:01	0,16
A*01:01	0,165	B*08:01	0,114	C*07:02	0,141
A*03:01	0,14	B*44:02	0,095	C*04:01	0,106
A*24:02	0,085	B*15:01	0,061	C*05:01	0,094
A*11:01	0,061	B*35:01	0,056	C*06:02	0,093
A*32:01	0,035	B*40:01	0,053	C*03:04	0,075
A*68:01	0,032	B*44:03	0,047	C*03:03	0,053
A*31:01	0,027	B*51:01	0,047	C*12:03	0,049
A*25:01	0,021	B*18:01	0,044	C*02:02	0,044
A*23:01	0,02	B*27:05	0,037	C*08:02	0,038
A*30:01	0,013	B*57:01	0,036	C*01:02	0,034
A*02:05	0,00966	B*13:02	0,024	C*15:02	0,022
A*30:02	0,009	B*55:01	0,019	C*12:02	0,00869
A*68:02	0,00838	B*35:03	0,016	C*03:02	0,00219
A*33:01	0,0081	B*49:01	0,016	C*08:01	0,00042
A*66:01	0,00409	B*37:01	0,014	C*17:01	0,00878
A*33:03	0,00321	B*40:02	0,013	C*15:04	0,00008
A*24:03	0,00193	B*14:01	0,01	C*12:04	0,00000
A*02:06	0,00182	B*35:02	0,01	**SUM:**	**0,92916**
A*74:01	0,0004	B*58:01	0,00726		
A*01:02	0,00011	B*56:01	0,00643		
A*02:07	0,00004	B*35:08	0,00435		
A*66:02	0,00003	B*41:01	0,00428		
A*02:03	0,00002	B*15:17	0,00363		
A*34:02	0,00092	B*53:01	0,00339		
A*24:07	0,00007	B*15:03	0,00137		
**SUM:**	**0,92278**	B*15:10	0,00038		
		B*58:02	0,00015		
		B*46:01	0,00008		
		B*54:01	0,00004		
		B*45:01	0,00589		
		B*48:01	0,0007		
		B*42:01	0,00028		
		B*81:01	0,00008		
		B*35:05	0,00004		
		B*51:06	0,00002		
		B*15:04	0,00001		
		B*35:11	0,00001		
		B*15:20	0,00000		
		**SUM:**	**0,88139**		

USA NMDP Chinese					
A		B		C	
Allele	Freqency	Allele	Freqency	Allele	Freqency
A*11:01	0,275	B*40:01	0,154	C*07:02	0,194
A*24:02	0,152	B*46:01	0,134	C*01:02	0,191
A*33:03	0,101	B*58:01	0,087	C*03:04	0,116
A*02:01	0,095	B*51:01	0,046	C*08:01	0,104
A*02:07	0,095	B*15:01	0,03	C*03:02	0,087
A*02:03	0,077	B*54:01	0,03	C*03:03	0,047
A*02:06	0,035	B*13:02	0,029	C*06:02	0,045
A*30:01	0,027	B*35:01	0,022	C*04:01	0,043
A*31:01	0,024	B*40:02	0,017	C*12:02	0,031
A*01:01	0,014	B*44:03	0,014	C*15:02	0,026
A*03:01	0,014	B*48:01	0,013	C*12:03	0,018
A*32:01	0,00618	B*07:02	0,0079	C*07:01	0,01
A*24:07	0,00446	B*35:03	0,00787	C*05:01	0,00362
A*68:01	0,00401	B*56:01	0,00609	C*02:02	0,00357
A*23:01	0,00209	B*37:01	0,00602	C*08:02	0,00121
A*02:05	0,00138	B*35:05	0,00542	C*17:01	0,00049
A*24:03	0,00138	B*57:01	0,00511	C*12:04	0,00004
A*74:01	0,00137	B*08:01	0,00463	C*15:04	0,00004
A*33:01	0,00055	B*27:05	0,00346	**SUM:**	**0,92097**
A*30:02	0,0003	B*18:01	0,00257		
A*66:01	0,00025	B*45:01	0,00203		
A*25:01	0,00018	B*81:01	0,00115		
A*68:02	0,00014	B*15:17	0,00078		
A*34:02	0,00002	B*55:01	0,00076		
A*66:02	0,00000	B*35:02	0,00072		
**SUM:**	**0,93131**	B*15:03	0,0007		
		B*49:01	0,00059		
		B*35:08	0,0005		
		B*41:01	0,00038		
		B*53:01	0,0003		
		B*14:01	0,00023		
		B*51:06	0,00015		
		B*15:10	0,00013		
		B*42:01	0,00007		
		B*58:02	0,00004		
		B*42:02	0,00003		
		B*35:11	0,00002		
		B*15:20	0,00001		
		**SUM:**	**0,63366**		

USA NMDP African American					
A		B		C	
Allele	Freqency	Allele	Freqency	Allele	Freqency
A*02:01	0,123	B*53:01	0,118	C*04:01	0,204
A*23:01	0,11	B*07:02	0,073	C*07:01	0,117
A*03:01	0,084	B*35:01	0,069	C*02:02	0,089
A*30:01	0,068	B*15:03	0,064	C*06:02	0,087
A*30:02	0,067	B*42:01	0,053	C*07:02	0,071
A*68:02	0,06	B*45:01	0,05	C*17:01	0,068
A*74:01	0,055	B*44:03	0,046	C*03:04	0,057
A*33:03	0,052	B*58:02	0,042	C*05:01	0,034
A*01:01	0,047	B*08:01	0,038	C*08:02	0,034
A*68:01	0,04	B*58:01	0,038	C*03:02	0,018
A*34:02	0,034	B*15:10	0,035	C*12:03	0,015
A*24:02	0,025	B*18:01	0,032	C*03:03	0,013
A*33:01	0,021	B*49:01	0,028	C*01:02	0,00776
A*02:05	0,015	B*51:01	0,022	C*15:02	0,00537
A*32:01	0,015	B*44:02	0,021	C*12:02	0,00115
A*11:01	0,014	B*81:01	0,02	C*08:01	0,00105
A*66:01	0,014	B*40:01	0,013	C*15:04	0,00001
A*31:01	0,01	B*15:01	0,011	C*12:04	0,00000
A*66:02	0,00886	B*27:05	0,00843	**SUM:**	**0,82234**
A*01:02	0,00416	B*14:01	0,00792		
A*25:01	0,00344	B*13:02	0,00782		
A*02:06	0,00071	B*57:01	0,00711		
A*24:07	0,00053	B*37:01	0,0055		
A*24:03	0,00041	B*15:17	0,00451		
A*02:03	0,00016	B*55:01	0,00399		
A*02:07	0,00002	B*40:02	0,00334		
**SUM:**	**0,87229**	B*41:01	0,00299		
		B*56:01	0,00234		
		B*35:03	0,00198		
		B*35:02	0,00084		
		B*48:01	0,00045		
		B*35:08	0,00039		
		B*35:05	0,00037		
		B*46:01	0,00007		
		B*15:04	0,00004		
		B*35:11	0,00004		
		B*54:01	0,00003		
		B*51:06	0,00002		
		B*15:20	0,00000		
		**SUM:**	**0,83118**		

USA NMDP Hispanic					
A		B		C	
Allele	Freqency	Allele	Freqency	Allele	Freqency
A*02:01	0,209	B*35:01	0,071	C*04:01	0,176
A*24:02	0,132	B*51:01	0,061	C*07:02	0,121
A*03:01	0,074	B*07:02	0,058	C*07:01	0,102
A*01:01	0,073	B*44:03	0,055	C*06:02	0,061
A*68:01	0,048	B*40:02	0,048	C*03:04	0,06
A*11:01	0,046	B*18:01	0,041	C*05:01	0,058
A*31:01	0,044	B*44:02	0,04	C*01:02	0,054
A*23:01	0,037	B*08:01	0,039	C*08:02	0,053
A*30:02	0,027	B*15:01	0,027	C*12:03	0,042
A*32:01	0,026	B*49:01	0,026	C*15:02	0,039
A*68:02	0,025	B*53:01	0,02	C*02:02	0,037
A*33:01	0,022	B*45:01	0,017	C*03:03	0,03
A*02:06	0,02	B*27:05	0,016	C*17:01	0,02
A*30:01	0,02	B*35:03	0,015	C*08:01	0,015
A*02:05	0,015	B*57:01	0,015	C*12:02	0,012
A*25:01	0,01	B*40:01	0,014	C*03:02	0,00494
A*33:03	0,00818	B*48:01	0,014	C*15:04	0,00011
A*74:01	0,00727	B*58:01	0,014	C*12:04	0,00004
A*66:01	0,00641	B*13:02	0,013	**SUM:**	**0,88509**
A*24:03	0,00492	B*15:03	0,013		
A*34:02	0,00465	B*35:02	0,013		
A*01:02	0,00139	B*41:01	0,01		
A*66:02	0,00079	B*55:01	0,00906		
A*24:07	0,0003	B*14:01	0,00892		
A*02:03	0,00016	B*37:01	0,00749		
A*02:07	0,00006	B*15:17	0,00677		
**SUM:**	**0,86213**	B*42:01	0,00677		
		B*35:08	0,006		
		B*35:05	0,00561		
		B*15:10	0,00551		
		B*15:04	0,00389		
		B*58:02	0,0037		
		B*56:01	0,00344		
		B*81:01	0,00229		
		B*35:11	0,0004		
		B*46:01	0,00023		
		B*15:20	0,00017		
		B*54:01	0,00009		
		B*51:06	0,00004		
		**SUM:**	**0,71038**		

USA NMDP Mexican or Chicano					
A		B		C	
Allele	Freqency	Allele	Freqency	Allele	Freqency
A*02:01	0,223	B*35:01	0,08	C*04:01	0,171
A*24:02	0,13	B*51:01	0,058	C*07:02	0,137
A*03:01	0,081	B*07:02	0,057	C*07:01	0,092
A*01:01	0,074	B*40:02	0,057	C*03:04	0,073
A*31:01	0,053	B*44:03	0,047	C*06:02	0,06
A*68:01	0,052	B*08:01	0,042	C*05:01	0,057
A*02:06	0,05	B*18:01	0,041	C*01:02	0,051
A*11:01	0,048	B*44:02	0,041	C*08:02	0,051
A*23:01	0,026	B*15:01	0,031	C*12:03	0,039
A*32:01	0,025	B*48:01	0,025	C*03:03	0,037
A*30:02	0,023	B*49:01	0,023	C*08:01	0,036
A*33:01	0,02	B*27:05	0,022	C*15:02	0,036
A*68:02	0,018	B*13:02	0,014	C*02:02	0,034
A*30:01	0,016	B*40:01	0,014	C*17:01	0,014
A*02:05	0,014	B*45:01	0,014	C*12:02	0,011
A*25:01	0,011	B*35:02	0,013	C*03:02	0,00372
A*33:03	0,00493	B*35:03	0,013	C*15:04	0,00021
A*66:01	0,00492	B*57:01	0,013	C*12:04	0,00006
A*74:01	0,00443	B*53:01	0,012	**SUM:**	**0,90299**
A*34:02	0,00254	B*15:03	0,01		
A*24:03	0,00169	B*41:01	0,00992		
A*01:02	0,00124	B*55:01	0,00903		
A*24:07	0,0004	B*58:01	0,00901		
A*66:02	0,00032	B*14:01	0,00874		
A*02:03	0,00012	B*37:01	0,0073		
A*02:07	0,00002	B*35:08	0,00523		
**SUM:**	**0,88461**	B*15:17	0,00454		
		B*56:01	0,0041		
		B*42:01	0,00341		
		B*58:02	0,00244		
		B*15:10	0,00242		
		B*35:05	0,00153		
		B*81:01	0,00126		
		B*15:04	0,00024		
		B*46:01	0,0002		
		B*35:11	0,00009		
		B*51:06	0,00006		
		B*54:01	0,00006		
		B*15:20	0,00001		
		**SUM:**	**0,69659**		

USA NMDP South Asian Indian					
A		B		C	
Allele	Freqency	Allele	Freqency	Allele	Freqency
A*01:01	0,155	B*51:01	0,075	C*06:02	0,139
A*11:01	0,14	B*44:03	0,074	C*04:01	0,136
A*24:02	0,136	B*35:03	0,072	C*07:02	0,108
A*33:03	0,099	B*57:01	0,068	C*15:02	0,108
A*68:01	0,068	B*35:01	0,062	C*07:01	0,104
A*03:01	0,064	B*07:02	0,044	C*12:02	0,081
A*02:01	0,049	B*58:01	0,042	C*12:03	0,049
A*32:01	0,039	B*08:01	0,037	C*03:02	0,042
A*31:01	0,033	B*37:01	0,034	C*01:02	0,035
A*02:06	0,018	B*18:01	0,025	C*08:01	0,028
A*30:01	0,017	B*40:01	0,022	C*03:04	0,016
A*02:03	0,011	B*55:01	0,02	C*03:03	0,015
A*24:07	0,011	B*13:02	0,018	C*02:02	0,00931
A*02:05	0,00998	B*15:01	0,016	C*05:01	0,00836
A*23:01	0,00664	B*15:17	0,014	C*17:01	0,00387
A*24:03	0,00599	B*27:05	0,00828	C*12:04	0,00269
A*30:02	0,00243	B*44:02	0,00816	C*08:02	0,00192
A*74:01	0,0015	B*35:02	0,00726	C*15:04	0,00032
A*33:01	0,00124	B*51:06	0,00709	**SUM:**	**0,88747**
A*02:07	0,0006	B*49:01	0,00614		
A*66:01	0,00034	B*56:01	0,00608		
A*68:02	0,00028	B*48:01	0,00368		
A*25:01	0,00013	B*41:01	0,00347		
A*34:02	0,00004	B*40:02	0,00296		
A*01:02	0,00001	B*35:08	0,00201		
A*66:02	0,00001	B*53:01	0,00126		
**SUM:**	**0,86919**	B*46:01	0,00102		
		B*45:01	0,00088		
		B*35:05	0,00058		
		B*54:01	0,00027		
		B*15:03	0,00023		
		B*15:10	0,0002		
		B*14:01	0,00019		
		B*42:01	0,00011		
		B*58:02	0,00006		
		B*15:04	0,00005		
		B*81:01	0,00005		
		B*35:11	0,00001		
		SUM:	0,68304		
		**SUM:**	**0,69659**		

Supplementary Table 6 Frequencies of HLA subtype coverage of the B-LCL cell line panel presented in Supplementary Table 3 in different ethnic groups

Source: www.allelefrequencies.net

Supplementary Table 7

ID	Name	Human		Mouse		AA difference	Protein identity
		UniProtID	Sequence	UniProtID	Sequence		
REF	CD20	P11836	SLFLGILSV	P19437	SVFLGILSA	2	74.83%
#1	CD6	P30203	WLFFGITGL	Q61003	WLFLGIAGL	2	70.29%
#2	S52A3	Q9NQ40	LLFLGVLSV	Q9D6X5	LLFLGVLTV	1	75.43%
#3	ARAID	Q6UW56	LMFFGILGA	Q6PGD0	LMFFGILGS	1	79.37%
#4	BTNL8	Q6UX41	GLFFGIVGL	No entry			
#5	TTMP	Q5BVD1	SIFLGVITV	Q8C5C9	TIFLCFIIV	4	67.28%
#6	S36A4	Q6YBV0	LVFIGIISV	Q8CH36	LVFIGIISV	0	88.20%
#7	T255A	Q5JRV8	GLFLGIITA	Q8BHW5	GLFLGIITA	0	95.42%
#8	SEM7A	O75326	SLWLGVLPT	Q9QUR8	SFWLGVLPT	1	89.44%
#9	LETMD1	Q6P1Q0	CLFLGIISI	Q924L1	CLFVGLISI	2	80.00%
#10	MSH3	P20585	NIFIGIVGV	P13705	NLSVGVVGV	4	80.66%
#11	AP5S1	Q9NUS5	VVWLGVLSL	Q9D742	VLWLGILSL	2	79.41%
#12	ABCA2	Q9BZC7	NLFIGITAT	P41234	NLFIGITAT	0	91.03%
#13	FETUA	P02765	HTFMGVVSL	P29699	HAFSPVASV	5	60.87%
#14	AL3A2	P51648	LTFLGIVAA	P47740	FVCLVAVAA	4	84.09%
#15	S35F6	Q8N357	SQWLGILAT	Q8VE96	SQWLGILIT	1	87.06%
#16	PAXI1	Q6ZW49	QIFFGITAC	Q6NZQ4	QIFFGLTAC	1	85.74%
#17	MRP2	Q92887	TCFLGIIST	Q8VI47	LCFFGIVST	3	77.58%
#18	CML1	Q99788	VCFLGILGN	P97468	VCFLGLLGN	1	80.32%
#19	O10G3	Q8NGC4	YIFLGVLSV	No entry			
#20	MARH6	O60337	FAWLGVVPL	Q6ZQ89	FAWLGVVPL	0	98.02%
#21	CNR1	P21554	MFWIGVTSV	P47746	MFWIGVTSV	0	97.25%
#22	CYAC3	Q8NBI2	HSWLGITTV	Q6P1H1	HSWLGITTV	0	84.71%
#23	POK18	Q9QC07	VSFLGVTTV	No entry			
#24	CHAD	O15335	GAFLGVTTL	O55226	AAFSGVTTL	2	92.74%
#25	T255B	Q8WV15	GSFLGIIGI	Q5FW56	PDHWPCCNH	9	61.09%
#26	O10R2	Q8NGX6	YFFLGILST	No entry			
#27	P4HA2	O15460	MAWFGVLSC	Q60716	MSWFGVLSW	2	91.78%
#28	SEM4F	O95754	GFFLGILAA	Q9Z123	GFFLGVLAA	1	91.17%
#29	GPR85	P60893	IAFLGVLSC	P60894	IAFLGVLSC	0	100.00%
#30	S15A3	Q8IY34	AAFFGVTAN	Q8BPX9	AAFFGVTSN	1	81.14%

Supplementary Table 7 Comparison between human and corresponding mouse sequences of investigated potential off-target epitopes of A23

Peptides in light brown induced response by A23-TCR-Ts only at high concentrations (n=3). Peptides in medium brown induced titratable responses by A23-TCR-Ts but processing of the epitope could not be confirmed (n=5). Peptides shown in dark brown were recognized by A23-TCR-Ts when transfected as minigenes in target cells (n=3). Blue, Amino acid sequences are identical in human and mouse. Red, Amino acids that differ between human and mouse.

SUPPLEMENTARY METHODS

Immunopeptidomics profiling of HLA mono allelic B721.221 cells

HLA class I-deficient B721.221 cells (IHW00001, FRED HUTCH Research Cell bank) were retrovirally transduced to express single HLA alleles[1] (HLA-A*01:01, HLA-A*02:01, HLA-A*03:01, HLA-A*11:01, HLA-A*24:02, HLA-B*07:02, HLA-B*08:01, HLA-B*40:01, HLA-B*44:02) and sorted (SH800 cell sorter, Sony) after staining with a PE anti-human HLA-A, B, C antibody (W632, Nordic Biosite AS, 311406). The cells were expanded to 100 x 10^6 and then lysed in 1 mL of lysis buffer (PBS containing 1% lauryl maltoside, 1 mM EDTA, 1 mM PMSF and 1:200 Sigma protease inhibitor) for 1 h on ice. HLA peptide complexes were purified by immunoprecipitation[2]. Briefly, the HLA peptide complexes were captured on to beads coated with pan HLA class I antibody, and the beads were then washed with 3mL each of 0.1 M Tris-HCl / 150 mM NaCl, 0.1 M Tris-HCl / 400 mM NaCl, again with 0.1 M Tris-HCl / 150 mM NaCl, and finally 0.1 M Tris-HCl. All peptide elutions were desalted with Discovery DSC-18 SPE column, vacuum concentrated and dissolved in 25 µL of 3% acetonitrile containing 0.1% TFA.

Cell lysis and protein digestion

Cells were lysed in lysis buffer (150 mM NaCl, 30 mM HEPES, pH 7.4, 1 mM EDTA, 2 mM MgCl2, 1% Lauryl maltoside (Merck; cat.no. 850520P-5G), protease inhibitor cocktail (Sigma; cat. no. P8340-5ML), 1 mM TCEP, 1 mM PMSF, 1 mM NaF, 1 mM Na3VO4, and 250 units of benzonase (Semba Biosciences; cat. no. R1006E)) for 1h on ice and centrifuged at 14,000g for 10 min. Protein (100 µg) from each cell type was then denatured and reduced with 0.3% SDS and 10 mM DTT (Merck; cat.no. D0632-5G) at 95^{0}C for 10 minutes and at RT in 35 minutes. Each sample was then alkylated with 30 mM Acrylamide (Merck; cat.no. A9099-25G) at RT in the dark for 45 minutes and quenched with 20mM DTT for 30 minutes at RT. 30 µl of Amine beads (Resyn Biosciences, cat.no. MR-AMN005) was washed 3 times with 70% acetonitrile (Merck; cat.no. 34851-1L) before adding to each protein sample. Acetonitrile was then added to each sample up to 70% final concentration. The beads were incubated for 1 hour at RT and washed with 95% acetonitrile, 80% ethanol, twice with deionized water, and resuspended in

100 µl of ammonium carbonate buffer containing 0.1% of proteasemax (Promega; cat.no. V2071). Trypsin (1.5 µg) was added to each sample and incubated at 1200 rpm overnight at 37 °C. 1% TFA final concentration was added to each sample to stop digestion and the peptides were purified similarly to the immunopeptidomics samples.

Mass spectrometry

The immunopeptidomics samples (5 µL) was analysed using an Ultimate 3000 nano-UHPLC system (Dionex, Sunnyvale, CA, USA) connected to a Q Exactive mass spectrometer (ThermoElectron, Bremen, Germany) equipped with a nano electrospray ion source[2]. A flow rate of 300 nL/min was employed with a solvent gradient of 3-35% B in 100 min, to 50% B in 13 min and then to 80% B in 2 min. The samples were also analysed using a longer solvent gradient of 3-35% B in 100 min, to 50% B in 13 min and then to 80% B in 2 min. Solvent A was 0.1% formic acid and solvent B was 0.1% formic acid / 90% acetonitrile. The mass spectrometer was operated in the data-dependent mode to automatically switch between MS and MS/MS acquisition. The method used allowed sequential isolation of up to the twelve most intense ions, depending on signal intensity (intensity threshold 1e5), for fragmentation using higher-energy collision induced dissociation (HCD) at a resolution R = 17,500 with NCE 27. The raw data was then analysed with PEAKS software (Bioinformatics Solutions Inc.). The tandem mass spectra were matched against the Uniprot Homo sapiens database. Precursor mass tolerance was set to 10 ppm, methionine oxidation was considered as variable modification, enzyme specificity was set to none and a product ion tolerance of 0.05 Da was used. Digested peptides were analyzed similarly to the immunopeptidomics samples, except for the total gradient of 90 minutes and the target ions already selected for MS/MS were dynamically excluded for 30 s. The raw data was then analyzed with Fragpipe v19.1 and MSfragger v3.7[3] using the default LFQ-MBR workflow. Parameters were set as follows: Alkylation at Cysteine as fixed modification; protein N-acetylation and methionine oxidation as variable modifications, and MBR was disabled. Trypsin without proline restriction enzyme

option was used, with two allowed miscleavages. The allowed false discovery rate was 0.01 (1%) for peptide and protein identification.

Quantitative PCR analysis

Total RNA was extracted from cells using the RNeasy Plus mini kit (Quiagen) and the RNA concentration was determined with the Qubit RNA BR Assay Kit (Thermo Fisher Scientific). Quantitative PCR (qPCR) was carried out using the KiCqStart One-Step Probe RT-qPCR ReadyMix kit (Sigma-Aldrich). The following TaqMan qPCR probes were used GAPDH Hs02758991_g1 /vic, B2M Hs00187842_m1, CD20 Hs00544819_m1, TTMP Hs01070716_m1, T255A Hs01075731_m1, LTMD1 Hs00374912_m1, NYESO-1 Hs00265824_m1, Ki67 Hs01032443_m1, FGRL1 Hs00222484_m1 (Thermo Fisher Scientific). 2^dCt method was employed for normalization and GAPDH or β_2 microglobulin were used as reference genes Fig. S5 A, B).

Retroviral gene transfer

Buffy coats from healthy blood donors were acquired form Oslo University Hospital's blood bank and PBMCs (peripheral blood mononuclear cells) were isolated using density gradient centrifugation. Fresh or cryopreserved PBMCs were seeded out at 1.3 x 10^6 cells/ml concentration on CD3 (OKT3, eBioscience, 16-0037-85) and CD28 (CD28.6, eBioscience, 16-0288-85) antibody coated plates and activated for 72 h. PBMCs were cultured in T-cell medium (TCM, CellGenix GMP DC Medium (CellGenix) supplemented with IL-7 and IL-15 (5 ng/ml each, PeproTech), 5% Normal Human Serum (Trina bioreactives) and 1% Penicillin/streptomycin). Retroviral supernatant was produced by plating 5 x 10^6 Phoenix-AMPHO retrovirus producer cells on 10 cm petri dishes (Day 1) for 24 hours and then transfecting them with 2.6 µg γ-retroviral vector DNA encoding A23 and 1G4 TCR sequences using X-tremeGENE 9 DNA Transfection reagent (Roche Diagnostics) according to the manufacturer`s instructions (Day 2). After 24 hours, the medium was refreshed and cells were placed to 32 ^oC for optimal virus production (Day 3). Virus supernatants were collected after 24 and 48 hours (Days 4 and

5). Activated PBMCs were collected, re-suspended in TCM at 0.5 10^6 cells/ml concentration, mixed with equal volume of retroviral supernatant, placed in non-tissue culture treated 6-well plates pre-coated with Retronectin (Takara) and spinoculated at 900 *g* for 60 min at 32 °C (Day 4 and 5). Transduction efficacy was determined on Day 8-10 using anti-mouse TCRβ chain antibody (PE, H57-597, BD Biosciences). Cells were cryopreserved on day 12-14. Before functional experiments, cells were rested for 48 h in TCM containing low concentration of cytokines (0.5 ng/ml of IL-7 and IL-15). Retroviral supernatants encoding full-length HLA alleles were produced as described above and used to transduce cell lines.

For murine T cell transduction, Ecotropic retroviruses encoding the A23 and DMF5 TCRs were obtained after transient transfection of Plat-E cells with 18 µg retroviral vector plasmid by calcium phosphate precipitation. 48 h after transfection, 3 ml of virus supernatant were harvested, filtrated and used for transduction. Spleen cells were isolated from HHD mice and erythrocytes were lysed by ammonium chloride treatment. Cells were incubated in splenocyte medium (SM, RPMI 1640, 10% FCS, 100 IU/ml penicillin-streptomycin, 1 mM sodium pyruvate, 1x non-essential amino acids, 50 µM 2-mercaptoethanol) supplemented with 1 µg/ml anti-mouse CD3, 0.1 µg/ml anti-mouse CD28 antibodies (both BioLegend, 145-2C11) and 10 IU/ml human IL-2 (Proleukin S) at a concentration of $2x10^6$/ml. On the following day, $1.5x10^6$ cells were transduced by spinoculation in 24-well non-tissue culture-treated plates pre-coated with 12.5 µg/ml Retronectin (TaKaRa) and virus particles (3200 x g, 90 min, 4 °C) in 1 ml SM supplemented with 10 IU/ml IL-2 and $4x10^5$ mouse T-Activator beads (Thermo Fisher Scientific). A second transduction was performed on the following day by spinoculation with 1 ml virus supernatant (+ 10 IU/ml IL-2). T cells were expanded in SM (+ 50 ng/ml IL-15 (Miltenyi Biotech) for 3 (ATT) or 10 days (co-cultures), respectively.

Protein detection by Western blot

10^6 cells were lysed in RIPA buffer, 4x LDS (Thermofisher) and 50mM DTT was then added to cleared lysate. Samples were loaded onto a NuPAGE 4-12% Bis-Tris gel (Invitrogen) and transferred to iBlot®2

PVDF MiniStacs (Invitrogen). 5%BSA/TBSt was as blocking buffer, with NY-ESO-1 monoclonal ab, clone E978 (Invitrogen) used as primary antibody at 1:1000. Polyclonal Rabbit anti-mouse immunoglobulin HRP (Dako) was added as secondary antibody at 1:20,000. β-actin HRP antibody, clone 7D2C10 (Proteintech) at 1:10,000 was used as a loading control. Proteins were visualized using Super Signal ™ West Dura Extended Duration Substrate (Thermo Scientific) and iBright 1500 (Invitrogen).

1. Strønen, E. *et al.* Targeting of cancer neoantigens with donor-derived T cell receptor repertoires. *Science (80-.).* **352**, 1337–1341 (2016).
2. Ali, M. *et al.* T cells targeted to TdT kill leukemic lymphoblasts while sparing normal lymphocytes. *Nat. Biotechnol.* **40**, 488–498 (2022).
3. Kong, A. T., Leprevost, F. V, Avtonomov, D. M., Mellacheruvu, D. & Nesvizhskii, A. I. msFragger: ultrafast and comprehensive peptide identification in mass spectrometry-based proteomics. **14**, 513 (2017).
