# Supplementary material for: A systematic safety pipeline for selection of T-cell receptors to enter clinical use
Source: NPJ Vaccines. 2023 Aug 22;8:126. doi: 10.1038/s41541-023-00713-y (PMC10444760; doi:10.1038/s41541-023-00713-y)
Supplement: Supplementary file 2 — Reporting Summary [file 41541_2023_713_MOESM2_ESM.pdf]

nature research

Corresponding author(s): Johanna Olweus
Matthias Leisegang

Last updated by author(s): 30/06/23

Reporting Summary

Nature Research wishes to improve the reproducibility of the work that we publish. This form provides structure for consistency and transparency in reporting. For further information on Nature Research policies, see our Editorial Policies and the Editorial Policy Checklist.

Statistics

For all statistical analyses, confirm that the following items are present in the figure legend, table legend, main text, or Methods section.

n/a	Confirmed	
☐	☒	The exact sample size (*n*) for each experimental group/condition, given as a discrete number and unit of measurement
☐	☒	A statement on whether measurements were taken from distinct samples or whether the same sample was measured repeatedly
☐	☒	The statistical test(s) used AND whether they are one- or two-sided<br>*Only common tests should be described solely by name; describe more complex techniques in the Methods section.*
☒	☐	A description of all covariates tested
☐	☒	A description of any assumptions or corrections, such as tests of normality and adjustment for multiple comparisons
☐	☒	A full description of the statistical parameters including central tendency (e.g. means) or other basic estimates (e.g. regression coefficient) AND variation (e.g. standard deviation) or associated estimates of uncertainty (e.g. confidence intervals)
☒	☐	For null hypothesis testing, the test statistic (e.g. *F*, *t*, *r*) with confidence intervals, effect sizes, degrees of freedom and *P* value noted<br>*Give P values as exact values whenever suitable.*
☒	☐	For Bayesian analysis, information on the choice of priors and Markov chain Monte Carlo settings
☒	☐	For hierarchical and complex designs, identification of the appropriate level for tests and full reporting of outcomes
☒	☐	Estimates of effect sizes (e.g. Cohen's *d*, Pearson's *r*), indicating how they were calculated

Our web collection on statistics for biologists contains articles on many of the points above.

Software and code

Policy information about availability of computer code

Data collection	1. Flow cytometry was performed on BD LSR II, MACSQuant or FACSCalibur flow cytometers and data was acquired with the help of BD FACSDIVA V8.0.1 software.<br>2.a Immunoprecipitation- targeted Mass spectrometry (IP-MS) data was acquired using an Ultimate 3000 nano-UHPLC system (Dionex, Sunnyvale, CA, USA) connected to a Q Exactive mass spectrometer (ThermoElectron, Bremen, Germany) equipped with a nano electrospray ion source.<br>2.b Mass-spectrometry was performed on a QExactive HF Orbitrap mass spectrometer coupled to an Easy-nLC1000 liquid chromatograph (both Thermo Fisher Scientific). The Easy-nLC1000 was equipped with a PepMap RSLCC18 column (Thermo Fisher Scientific; cat. no. ES903, 2µm, 100Å, 75µmx50 cm). Water with 0.1% formic acid was used as solvent A, and acetonitrile with 0.1% formic acid was used as solvent B.
Data analysis	1. Flow cytometry data were analyzed with FlowJo versions 9 and 10.<br>2. Numerical data were statistically analyzed and graphs were generated with the help of GraphPad Prism version 8 software.<br>3. Sequence logos were produced by Seq2Logo<br>4. Results of mimotope screens were probed against the curated human proteome databases UniProtKB/Swiss-Prot and Protein Data Bank using the ScanProsite tool to identify potentially cross-reactive proteins.<br>5. HLA-A*02:01 binding affinity and Rank was predicted by NetMHC 4.0.<br>6. Peptide-MHC complex models were visualized by the PyMOL molecular visualization system. Protein data bank file for 1G4 TCR: 2BNR<br>7.<br>7a For IP-MS, the raw data was then analysed with PEAKS software (Bioinformatics Solutions Inc.). The tandem mass spectra were matched against the Uniprot Homo sapiens database.<br>7b For MS, the raw data was then analyzed with Fragpipe v19.1 and MSfragger v3.7 (https://doi.org/10.1038/nmeth.4256). The tandem mass spectra were matched against the Uniprot Homo sapiens database using the default LFQ-MBR workflow.

For manuscripts utilizing custom algorithms or software that are central to the research but not yet described in published literature, software must be made available to editors and reviewers. We strongly encourage code deposition in a community repository (e.g. GitHub). See the Nature Research guidelines for submitting code & software for further information.

Data

Policy information about availability of data

All manuscripts must include a data availability statement. This statement should provide the following information, where applicable:

- Accession codes, unique identifiers, or web links for publicly available datasets
- A list of figures that have associated raw data
- A description of any restrictions on data availability

The data that support the findings of this study are included in the manuscript and in supplementary information. Source data are also provided. Additionally, datasets used in the study are: Protein Data Bank (accession code: 2BNR), curated human proteome databases UniProtKB/Swiss-Prot and Protein Data Bank by ScanProsite tool (https://prosite.expasy.org/scanprosite/), peptide-MHC class I binding prediction algorithm NetMHC version 4.0 (http://www.cbs.dtu.dk/services/NetMHC/)

Field-specific reporting

Please select the one below that is the best fit for your research. If you are not sure, read the appropriate sections before making your selection.

☒ Life sciences ☐ Behavioural & social sciences ☐ Ecological, evolutionary & environmental sciences

For a reference copy of the document with all sections, see nature.com/documents/nr-reporting-summary-flat.pdf

Life sciences study design

All studies must disclose on these points even when the disclosure is negative.

Sample size	Sample sizes were chosen so that the multiple steps should be possible to perform by a single staff member, facilitating wide-spread use in laboratories developing TCRs for clinical applications. Sample sizes were chosen to In animal experiments samples sizes were expected to be sufficient based upon our previous observations with cell line based HHD animals.
Data exclusions	Animals were excluded from analysis if they died due to reasons unrelated to tumor burden or unrelated to T-cell therapy.
Replication	Replication of experiments is described in detail in all figure legends. Briefly, functional tests were repeated in three independent experiments and pooled and pooled data is shown in the manuscript. Functional analysis was performed with two independent methods with different readouts (i) ELISA (IFN-gamma secretion) and (ii) flow cytometric measurements of T cell activation by CD137 upregulation. Results from both T-cell functional readouts correlated strongly with each other, as shown in the manuscript in Supplementary figures S2c and S3b.<br>Figure 4 shows one representative experiment of two performed with similar results with all three technical replicates displayed as circles. Tumor volume measurement data was pooled from 3 experiments. Number of animals included are marked on the figures.
Randomization	Mice were randomized into experimental groups so that mean tumor size was comparable among groups. On the day of T-cell transfer, mice were ranked by tumor size and sequentially allocated to treatment groups to ensure equal average tumor sizes between groups.
Blinding	Examiners were not blinded to treatment groups. Mice were sacrificed when the tumors reached the maximum permitted size or if the overall health-condition of animals was poor due to tumor burden. Blinding would be impractical due to the limitations related to manpower, and because of the potential treatment-related side-effects that needed to be closely assessed. Due to the nature of the other in vitro experiments, blinding was not possible and is not generally performed in the field as data acquisition is quantitative (flow cytometry or MS) rather than qualitative, and therefore less influenced by observer bias. Histological sections were evaluated by a pathologist in a blinded manner.

Reporting for specific materials, systems and methods

We require information from authors about some types of materials, experimental systems and methods used in many studies. Here, indicate whether each material, system or method listed is relevant to your study. If you are not sure if a list item applies to your research, read the appropriate section before selecting a response.

Materials & experimental systems

n/a	Involved in the study	
☐	☒	Antibodies
☐	☒	Eukaryotic cell lines
☒	☐	Palaeontology and archaeology
☐	☒	Animals and other organisms
☒	☐	Human research participants
☒	☐	Clinical data
☒	☐	Dual use research of concern

Methods

n/a	Involved in the study	
☒	☐	ChIP-seq
☐	☒	Flow cytometry
☒	☐	MRI-based neuroimaging

Antibodies

Antibodies used

The following fluorescently labelled antibodies were used: anti-human - CD8a (FITC or Brilliant Violet 421, RPA-T8, BioLegend), anti-human - CD137 (Alexa Fluor 647, 4B4-1, Thermo Fisher Scientific), anti-human - HLA-A:02*01 (PE or Alexa Fluor 647, BB7.2, BioLegend) anti-mouse TCRβ chain (PE, H57-597, BD Biosciences), anti-mouse - CD3 (APC, 145-2C11, BioLegend), anti-mouse - CD8 (Brilliant Violet 421, 53-6.7, BioLegend) and isotype control antibody (mouse IgG2b, Alexa Fluor 647, AbD Serotec). In some cases, anti-human TCRvβ-specific antibodies (A23: TCRvβ17 clone E17.5F3.15.13 PE from Beckman Coulter) or chimeric A2/Kb pentamers (DMF5 TCR) loaded with ELAGIGILTV peptide (PE, ProImmune) were used to analyse the expression of TCRs. In some experiments, Cell Trace Violet (CTV, Thermo Fisher Scientific) labelling was used to distinguish between target and effector cells. Live/Dead Fixable Near-IR Dead Cell Stain kit or SYTOX Blue (both Thermo Fisher Scientific) were used to exclude dead cells in flow cytometry experiments.

Validation

All antibodies used in the study are available commercially and have been validated by commercial suppliers for use in diagnostics/research on samples generated from humans or mice, and for flow cytometry or ELISA as applicable.

Eukaryotic cell lines

Policy information about cell lines

Cell line source(s)

The following cell lines were obtained from American Type Culture Collection (ATCC) and German Collection of Microorganisms and Cell Cultures (DSMZ): K562 (CCL-243), JVM-2 (CRL-3002), OCI-M2 (ACC 619), HEP G2 (ACC 180), U-2 OS (HTB-96), U-87 MG (HTB-14), A-431 (CRL-1555), HCT 116 (CCL-247), Jurkat (TIB-152), NALM-6 (CRL-3273), Phoenix-Ampho (CRL-3213), U266 (ACC 9). HLA-typed lymphoblastoid cell lines (LCLs) covering the most frequent HLA alleles across multiple ethnicities were purchased from Fred Hutchinson Cancer Center and Merck and listed in Table S3.

Authentication

Authenticated cell lines were purchased from ATCC, DSMZ, Fred Hutchinson Cancer Center and Merck and cryopreserved in aliquots labeled according to passage. Only low passages (1-4 passages) were used to start cultures. The identity of the passage used (5 or higher) experimentally of the cell lines was ascertained by short tandem repeat DNA profiling, a service provided by Labcorp DNA Identification Lab, NC, USA (formerly Genetica, https://celllineauthentication.com/). An in-house immortalized HLA-A02*01positive B-LCL line wasregularly tested for CD20 staining to confirm their B cell origin.

Mycoplasma contamination

Cell lines were cultured as instructed by the manufacturers, were regularly tested for mycoplasma contamination (MycoAlert PLUS Mycoplasma Detection Kit, LT07-318). Cells were confirmed negative before experimental use.

Commonly misidentified lines
(See ICLAC register)

No commonly misidentified cells were used in the study

Animals and other organisms

Policy information about studies involving animals; ARRIVE guidelines recommended for reporting animal research

Laboratory animals

3-5x10 million MC703-SLF or MC703/HHD-SLF cells were subcutaneously injected in 100 µl PBS into the left flank of HHDxRag1-/- mice (12-20 weeks old, female or male). Tumor growth was analysed 2-3 times a week by determination of tumor volume using caliper measurements according to π/6 x (abc). Mice were randomized into experimental groups so that mean tumor size was comparable among groups. On the day of T-cell transfer, mice were ranked by tumor size and sequentially allocated to treatment groups to ensure equal average tumor sizes between groups. Mice were treated with HHD TCR-Ts earliest 3-4 weeks after tumor cell injection. HHD TCR-Ts were analyzed for expression of CD8, A23 (TCRvβ17), and DMF5 (A2/Kb:ELA) by flow cytometry and intravenously injected in 100 µl PBS (adjusted to 1x106 CD8+TCR+ HHD T cells per mouse) 3 days after transduction. Examiners were not blinded to treatment groups. Mice were sacrificed when the tumors reached the maximum permitted size or if the overall health-condition of animals was poor due to tumor burden. Animals were excluded from analysis if they died due to reasons unrelated to tumor burden or T-cell therapy.

Wild animals

No wild animals were used.

Field-collected samples

No field-collected samples were used.

Ethics oversight

Animal experiments were approved by the German State Office for Health and Social Affairs (Landesamt für Gesundheit und Soziales). All experiments were performed in compliance with the institutional guidelines and 2010/63/EU directive on the protection of animals used for scientific purposes.

Note that full information on the approval of the study protocol must also be provided in the manuscript.

Flow Cytometry

Plots

Confirm that:

☒ The axis labels state the marker and fluorochrome used (e.g. CD4-FITC).

☒ The axis scales are clearly visible. Include numbers along axes only for bottom left plot of group (a 'group' is an analysis of identical markers).

☐ All plots are contour plots with outliers or pseudocolor plots.

☒ A numerical value for number of cells or percentage (with statistics) is provided.

Methodology

Sample preparation	Samples analyzed by flow cytometry contained mononuclear cells in suspension isolated from buffy coats of healthy blood donors by density gradient centrifugation that were cultured with standard media as detailed in the manuscript. Samples were then cryopreserved and stored in liquid nitrogen in designated cell biobanks in our institutions before use in the experiments as detailed in the methods. Cell lines utilized were cultured for variable amounts of time in recommended media prior to experiments.<br>Blood and tissue samples were harvested from HHD mice. For flow cytometry RBC lysis was performed by ACK lysis buffer, followed by washing and surface or intracellular staining with different antibodies for flow cytometry analysis.
Instrument	Flow cytometry was performed on a BD LSR II, MACSQuant or FACSCalibur flow cytometers (BD Biosciences) For Western Blot Proteins were visualized using Super Signal ™ West Dura Extended Duration Substrate (Thermo Scientific) and iBright 1500 (Invitrogen)
Software	For data collection for all experiments, BD FACSDiva V8.0.1 was used. For data analysis FlowJo versions 9 and 10 were used.
Cell population abundance	HLA A-2 negative tumor cell lines that were transduced with HLA-A2 and purified by FACS (purity > 95%).<br>When using cell lines expressing RNA constructs encoding 30 AA long sections containing a potentially cross-reactive 9 AA long peptide in the middle a green fluorescent protein (GFP) tag was used to control for transfection efficiency as shown in Supplementary Fig. S4.<br>For human cells, transduction efficacy was determined on Day 8-10 post transduction using anti-mouse TCRβ chain antibody. Only cultures with >60% of transduced CD8+ cells were included in functional tests.<br>In murine cells) anti-human TCRvβ-specific antibodies (A23: TCRvβ17 clone E17.5F3.15.13 PE from Beckman Coulter) or chimeric A2/Kb pentamers (DMF5 TCR) loaded with ELAGIGILTV peptide (PE, ProImmune) were used to analyse the expression of TCRs.
Gating strategy	For all flow cytometry experiments FSC-A/SSC-A was used for gating of mononuclear cells. Doublets were excluded. Live/Dead Fixable Near-IR Dead Cell Stain kit or SYTOX Blue -positive cells were gated out to exclude non-viable cells.<br><br>For flow cytometry-based cytotoxicity assays and T cell activation assays, tumor cell lines and transduced T cells were stained with surface antibodies and were gated as detailed in the methods and shown in Supplementary figure S2A. In some experiments, effector cells in the same well were pre-labeled with CellTrace Violet (CTV, Life Technologies) for separation from live target cells.<br><br>To analyze presence of transduced T cells in blood of HHD mice, single live cells were gated as described in methods and shown in Supplementary figure S9D. CD3+ CD8+ , human TCR-transduced T cells were identified as anti-human CD3+, CD8+ and TCRvβ17+.

☒ Tick this box to confirm that a figure exemplifying the gating strategy is provided in the Supplementary Information.
